# Mechanisms of chromosomal DNA replication in *Escherichia coli* and *Bacillus subtilis*

**DOI:** 10.1093/femsre/fuag021

**Published:** 2026-05-14

**Authors:** Rubén Torres, Begoña Carrasco, Silvia Ayora, Juan C Alonso

**Affiliations:** Department of Microbial Biotechnology, Centro Nacional de Biotecnología, CNB-CSIC, 28049 Madrid, Spain; Department of Microbial Biotechnology, Centro Nacional de Biotecnología, CNB-CSIC, 28049 Madrid, Spain; Department of Microbial Biotechnology, Centro Nacional de Biotecnología, CNB-CSIC, 28049 Madrid, Spain; Department of Microbial Biotechnology, Centro Nacional de Biotecnología, CNB-CSIC, 28049 Madrid, Spain

**Keywords:** DNA replication, replisome, DNA polymerases, translesion synthesis, replication restart

## Abstract

Faithful DNA replication, which is a highly orchestrated process, is essential in all living organisms to ensure accurate transmission of genetic information to their descendants. In this review, we summarize the molecular mechanisms and dynamics of DNA replication in *Escherichia coli* and compare them with those of the phylogenetically distant *Bacillus subtilis*. Although the central features of replication initiation, elongation, termination, and restart are broadly conserved, distinct mechanisms have evolved to adapt each bacterium to its complex environment. This review highlights the players and outlines both the shared and divergent molecular mechanisms governing how the multi-component replication machines, the replisomes, are assembled at the origin of chromosomal replication, *oriC*, on a circular genome, undergo bidirectional replication elongation, and are ultimately disassembled upon reaching the terminus of replication, *ter*, region. In response to stress, (a) replication restart mechanism(s) operating at sites other than *oriC* re-assemble the replisome, allowing unidirectional DNA synthesis to resume, thereby ensuring completion of the cell cycle and maintenance of cell viability.

## Teaser

This review compares the DNA replication process in the two model bacteria *Escherichia coli* and *Bacillus subtilis*, emphasizing the similarities and differences in the initiation, elongation, and termination stages, as well as exploring how these bacteria restart replication at sites other than oriC.

## Introduction

Accurate, efficient, and complete genome duplication of double-stranded DNA (dsDNA) is essential for the faithful transmission of genetic information to the next generation, with rare errors giving rise to beneficial or deleterious genetic variation. In bacteria with circular chromosomes, replication proceeds bidirectionally from the *cis*-acting chromosomal replication initiation origin (*oriC*) and is tightly regulated by different mechanisms at the pre-initiation, initiation, transition to elongation, and termination stages. During replication elongation, the antiparallel orientation of DNA strands and the unidirectional 5′→3′ activity of DNA polymerases (DNAP) necessitate coordinated semiconservative synthesis of the two parental strands. As a result, the leading strand is synthesized continuously, whereas the lagging strand is synthesized discontinuously. Replication terminates at the *cis*-acting replication terminus (*ter*) zone opposite *oriC*. Bacteria have evolved specific mechanisms to overcome challenges encountered on the DNA template and to reinitiate unidirectional replication outside *oriC* when replication is perturbed by endogenous or exogenous threats.

Genetic, cytological, and biochemical studies -including reconstituted *in vitro* replication systems developed ∼30 years ago- as well as single-molecule and structural studies, have helped to define the components of DNA synthesis in *Escherichia coli*, the best-characterized bacterium of the Proteobacteria (*a.k.a*. Pseudomonadota) Phylum (reviewed in Lazowski et al. 2024), and considered as the most important Gram-negative model organism. *Escherichia coli* is an aerobic and facultative anaerobic organism commonly found in the lower intestine and urinary tract. Nevertheless, the replication machinery varies among phyla and, in some cases, among classes, because bacteria face diverse complex mechanistic challenges and environments, and have evolved distinct replication strategies. To highlight those diverse mechanisms of DNA synthesis, we focus on *Bacillus subtilis*, the best-characterized bacterium of the Firmicutes (*a.k.a*. Bacillota) Phylum, for which a fully functional multi-protein replisome has also been reconstituted from purified proteins ∼16 year ago (reviewed in Murray et al. 2017). *Bacillus subtilis* is the most common Gram-positive model organism. It is an aerobic soil bacterium that is also adapted to life in the intestinal tract and the rhizosphere. These two model bacteria, which are free-living, rod-shaped organisms equipped with specialized mechanistic complexes to replicate their genomes in different environments, are evolutionary separated by >2000 million years (Torres et al. 2025).

In this review, we compare the molecular mechanisms of replisome assembly at *oriC*, bidirectional replication elongation, and termination upon reaching the *ter* zone in *E. coli* with those in *B. subtilis*, highlighting species-specific differences that contribute to successful replication. We focus on proteins that facilitate and regulate the activity of DnaA to destabilize both DNA strands at a discrete region of *oriC*, on the poorly conserved mechanisms for loading the replicative DNA helicase that unwinds the parental strands from *oriC*, and on subsequent steps in which the recruited helicase promotes the loading of enzyme(s) required for primer synthesis and contributes to replisome assembly. The structure of the replisome in both model bacteria and the progression of replication elongation are summarized and compared. For instance, *E. coli* encodes a single essential replicative C-family DNA polymerase (DNAP) whereas *B. subtilis* possesses two essential replicative C-family enzymes (Dervyn et al. 2001, Kornberg and Baker 2005, O’Donnell 2006, McHenry 2011, Timinskas et al. 2014). The dynamics and stability of the replisome during replication elongation are discussed. Elongating replisomes eventually converge, leading to the accumulation of positive (+) supercoils between them. Replication through protein-bound *ter* sites coordinates the disassemble of replisomes approaching from opposite directions (Kornberg and Baker 2005).

In both model bacteria, endogenous factors that interfere with DNA replication -including misincorporated ribonucleoside monophosphates, oxidized nucleobases generated during cellular metabolism, nucleotide imbalance, collisions between the replication and transcription machineries, RNA-DNA hybrid structures (R-loops), among others- cause spontaneous replication stress and replication fork (RF) pausing. Likewise, external genotoxic agents -including UV irradiation, 4-nitroquinaline-1-oxide, methyl methanesulfonate, *etc*.- cause damage-induced replication stress. These endo- and exogenous threats stall RFs, causing either transient disassembly and barrier skipping, leaving a gap behind (as in the majority of cases in *E. coli*), or RF disassembly followed by repair before restart (as in *B. subtilis*). In the former case, lesions/barriers located distal to the replisome are circumvented or bypassed by different DNA damage tolerance (DDT) sub-pathways acting in a post-replicative manner, whereas in the latter case, DDT sub-pathways act directly at the stalled fork. A detailed description of how cells overcome endogenous or environmental threats that affect DNA replication is beyond the scope of this review and we refer readers to recent reviews (Michel and Sandler 2017, Marians 2018, Windgassen et al. 2018, Cox et al. 2023, Carrasco et al. 2024). In this review, however, we describe how both model bacteria use distinct sets of pre-primosomal proteins that act independently of *oriC* for unidirectional replisome reassembly at stalled forks.

The pivotal players in DNA replication in *E. coli* and *B. subtilis* are highlighted in Table 1. As described, some essential proteins are absent in one bacterium, some functions are essential in one species but not in the other, and some alternative pathway(s) is(are) present in only one of the two. Finally, we note that significant progress has been made in past years in understanding the diverse mechanisms of DNA replication initiation in other bacteria. These advances merit a dedicated analysis, and similarities and differences relative to the mechanisms described here will be compared in a separate review.

**Table 1:** Genes involved in canonical replication initiation, elongation, termination, and re-initiation in *E. coli* and *B. subtilis* (functional orthologs/analogs/counterparts).

Activity	*Escherichia coli*	*Bacillus subtilis*	Role of gene product
Initiator	*dnaA*	*dnaA*	*oriC*-recognition, replication initiation
Initiation remodelers	*ihf* ^a^	No	NAP, helps DnaA·ATP to melt DUE
	*hupAB* ^a^	*hbs*	NAP, helps DnaA·ATP to melt DUE
Helicase loaders	*No*	*dnaD*	contributes to helicase loading
	*No*	*dnaB*	contributes to helicase loading
	*dnaC*	*dnaI*	helicase loader
Primosome	*dnaB*	*dnaC*	5'→3' replicative DNA helicase, protein hub
	*dnaG*	*dnaG*	DNA primase, helicase remodeler
	No	*dnaE* ^b^	C-family DNAP, adds few dNMPs to RNA
ssDNA binding	*ssb*	*ssbA*	ssDNA binding, protein hub
DNAP HE	*dnaE* ^b^*-dnaQ-holE* ^a^	*polC* ^b^	DNAP core enzyme
	*dnaXZ/*γ^a,c,d^	No	clamp loader subunit
	*dnaX*/τ^c^	*dnaX*	clamp loader subunit
	*holA*/δ^c^	*holA*	clamp loader subunit
	*holB*/δ'^c^	*holB*	clamp loader subunit
	*holC*/χ^a,c^	No	clamp loader subunit
	*holD*/ψ^a,c^	No	clamp loader subunit
	*dnaN*/β	*dnaN*	processivity sliding-clamp
Accessory DNAPs	*polA*	*polA* ^a,e^?	A-family DNAP, Pol I, 3'→5' proofreading
	*polB* ^a^	No	B-family DNAP, Pol II, 3'→5' proofreading
Accessory helicases	*rep* ^a^ (*uvrD* ^a^ in Δ*rep*)	*pcrA*?	accessory 3'→5' DNA helicase
	No	*recD2* ^a^	accessory 5'→3' DNA helicase
Primer maturation	*polA*	*polA* ^a,f^	DNAP Pol I, endonuclease/5'→3'-exonuclease
	No	*fenA* (*ypcP*)^a,f^	flap endonuclease/5'→3'-exonuclease
	*rnhA* ^a^	*rnhC* ^a,f^	ribonuclease, degrades RNA-DNA hybrids
DNA topology	*topA* ^a^	*topA*	Topo I, nicks and rotates the DNA strands
	*topB* ^a^	*topB* ^a,g^	Topo III, decatenase
	*gyrAB*	*gyrAB*	Topo II, cleaves and rotates both strands
	*parCE*	*parCE* ^h^	Topo IV, cleaves and rotates both strands
Termination	*tus* ^a^	*rtp* ^a^	Replication termination at *ter* sites
TLS DNAP	*dinB* ^a^	*polY1* (*yqjH*)^a^	Y-family, Pol IV/PolY1
	*umuC* ^a^ and *umuD* ^a^	*polY2* (*yqjW*)^a,i^	Y-family, Pol V/PolY2
	No	*polA-polY1* ^a^	A- and Y-families, bipartite TLS DNAP
	No	*polA-polY2* ^a^	A- and Y-families, bipartite TLS DNAP
	No	*dnaE* ^b^?	C-family DNAP, primosome component
Replication re-start^j^	No	*priA-dnaD-dnaB*	pre-primosome complex, loads DnaC-DnaI
	*priA* ^a^-*priB* ^a^-*dnaT* ^a^	No	pre-primosome complex, loads DnaB-DnaC
	*priA* ^a^-*priC* ^a^-*dnaT* ^a^	No	pre-primosome complex, loads DnaB-DnaC
	*priC* ^a^-*rep* ^a^	No	pre-primosome complex, loads DnaB-DnaC

a Non-essential function.

b In *B. subtilis*, DnaE (DnaE3) is an error-prone DNAP that elongates the short RNA primer to create a hybrid RNA-DNA primer and PolC is the replicative core enzyme (Sanders et al. 2010), whereas in *E. coli*, DnaE/α (DnaE1), in concert with DnaQ/ε and HolE/θ forms the Pol III core enzyme (Kornberg and Baker 2005).

c In *E. coli*, DNAP subunits are denoted by Greek letters, but this nomenclature is not conserved in *B. subtilis*.

d In *E. coli*, a translational frameshift in the *dnaX* gene leads to the short DnaXZ/γ and the full length DnaX/τ protein, whereas only full-length DnaX is present in *B. subtilis*.

e *Bacillus subtilis* PolA lacks an active 3′→5′ proofreading domain.

f Primer maturation in *B. subtilis* is manly performed by FenA.

g *Bacillus subtilis* TopB may lack decatenation activity.

h Increased expression of the ParCE enzyme is necessary and sufficient to compensate for the absence of Topo I and Topo III enzymes.

i PolY2, which shares homology with the UmuC subunit of Pol V, may function as a standalone TLS DNAP.

j PriA is the only ubiquitous replication re-start protein. “No” denotes the absence of a counterpart in the indicated organism.

### Replication initiation

**Figure 1:**
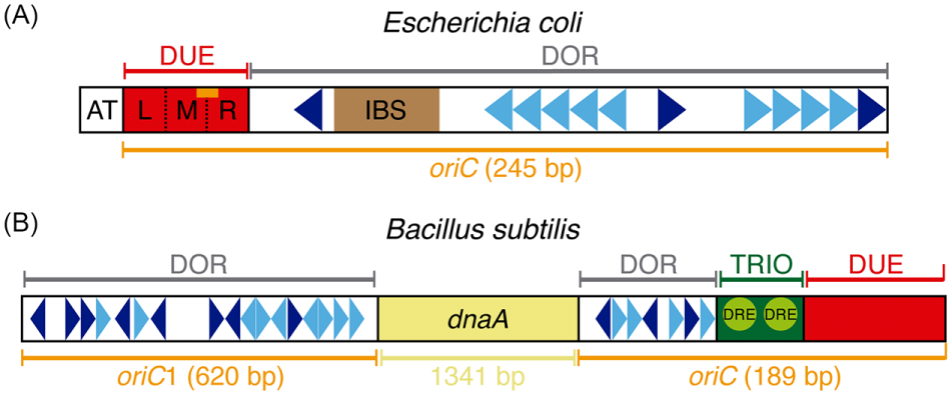
Schematic representation of the *oriC* regions in *E. coli* (A) and *B. subtilis* (B). (A) The 245 bp *oriC* consists of the DNA unwinding element (DUE) and DnaA oligomerization (DOR) regions. The DOR includes high-affinity DnaA boxes (dark blue arrowheads), low-affinity DnaA boxes (light blue arrowheads), and the IHF binding site (IBS). The DUE is divided into three regions of AT-rich 13-mer repeats (L, M, and R) and additionally contains putative DnaA-trio motifs (orange rectangle). An additional AT-rich region flanking the L sequence assists DUE unwinding. (B) The *oriC*1 and *oriC* regions comprise two DOR regions located up and downstream the *dnaA* gene, along with the DUE and the DnaA-trio (TRIO) motif. The DORs contain high-affinity DnaA boxes (zero or one mismatch; dark blue arrowheads) and low-affinity DnaA boxes (two or more mismatches; light blue arrowheads). The TRIO motif encompasses the DnaD recognition element (DRE). (A, B) Diagrams are not drawn to scale.

Bacteria with covalently closed circular genomes contain a single unit of DNA replication (a replicon) that initiates at a specific cis-acting replicator sequence (*oriC*) by the binding of a *trans*-acting initiator protein (Jacob and Brenner 1963). Both model bacteria possess genomes of roughly similar size and gene number. The central features of replication initiation are broadly conserved in both organisms. The timing and location of replisome assembly are determined by origin firing at a unique *oriC* region (Cairns 1963, Yoshikawa and Sueoka 1963): *E. coli* recognizes and initiates replication from a single *oriC* site located distal to the *dnaA* gene, whereas *B. subtilis* recognizes a bipartite *oriC* (composed of *oriC*1 and *oriC2*) located up- and downstream of the *dnaA* gene, and initiates strand separation and replication from a single site, *oriC2* (or simply *oriC*) (Fig. 1) (Ogasawara et al. 1985, Bramhill and Kornberg 1988). In both bacteria, the ubiquitous initiator DnaA in the ATP-bound form (DnaA·ATP), which is the active form for replication initiation, together with nucleoid-associated proteins, recognizes and “melts” parental DNA strands at *oriC* to initiate DNA replication (reviewed in Chodavarapu and Kaguni 2016, Jameson and Wilkinson 2017, and references therein). Overproduction of *E. coli* DnaA resulted in a decrease of the initiation mass and enhanced replication initiation in exponential cultures, conversely over-initiation of replication was not observed when *B. subtilis* DnaA was ∼10-fold overproduced in otherwise wt cells (Lobner-Olesen et al. 1989, Moriya et al. 1990).

DNA replication initiation is primarily regulated by controlling the levels and/or activity of DnaA. In *E. coli*, DnaA interacts directly with the replicative helicase (DnaB) to facilitate its loading, whereas in *B. subtilis*, DnaA recruits two additional proteins, DnaD and DnaB, which are required for loading the replicative helicase (DnaC). In both cases, two replicative DNA helicases are loaded in opposite orientations at the unwound region of *oriC*, one on each complementary strand, thereby allowing bidirectional replication and defining the lagging-strand template (Messer 2002, Chodavarapu et al. 2016, Blaine et al. 2023). Each assembled helicase then facilitates replisome recruitment by interacting with and loading the remaining component(s) of the primosome -the DnaG primase in *E. coli*, and both the DnaG primase and the DnaE DNAP in *B. subtilis*- as well as with the DnaX/τ subunit (the slash between DnaX and τ denotes alternative names in *E. coli*) of the clamp loader complex (CLC). The CLC, in turn, interacts with and loads the DnaN/β sliding-clamp and recruits two replicative DNAP holoenzymes (HE) (Chodavarapu et al. 2016, Blaine et al. 2023). As a result, two replisome complexes are assembled to synthesize each half of the chromosome by moving in opposite orientations (Ogasawara et al. 1985, Messer 2002, Lazowski et al. 2024).

### Replication initiation in *E. coli*

At *oriC*, DnaA·ATP occupies several specific 9-mer sequence motifs (termed DnaA-boxes, collectively forming the DnaA oligomerization region, DOR) as soon as they become accessible, usually after *oriC* DNA has been replicated (Fig. 1A) (Zyskind and Smith 1986, Speck et al. 1997). First, DnaA occupies the three highest-affinity sites, and this binding pattern persists throughout much of the cell cycle (Samitt et al. 1989). DNA replication initiates when the remaining lower-affinity DnaA-boxes in *oriC* are filled and a discrete topological structure is achieved, resulting from active transcription away from *oriC*, binding of nucleoid-associated proteins to *oriC*, and a certain degree of negative (−) supercoiling (Messer 2002, Katayama et al. 2017).

The mechanism by which DnaA melts the *oriC* region, as well as its disassembly from DnaA-boxes once DNA replication has initiated, has been under investigation for more than 30 years and is still not fully understood. The structure of the DnaA oligomer associated with *oriC* has not been determined, but magnetic tweezers experiments suggest that the DnaA·ATP-*oriC* complex adopts a right-handed helical conformation (Zorman et al. 2012). The specialized nucleoid-associated protein IHF (which recognizes and binds a specific sequence, IBS) or HU (which binds DNA with no sequence specificity) introduces a sharp DNA bend that facilitates DnaA·ATP ssDNA binding as a large oligomeric complex and full occupancy of duplex DNA at *oriC*, as well as melting of the adjacent DNA unwinding element (DUE), which contains three AT-rich sub-motifs (Fig. 1A) (Bramhill and Kornberg 1988, Ryan et al. 2002, Yoshida et al. 2023).

Bioinformatic analyses have identified three copies of a trinucleotide DnaA target-like sequence (termed KAK-boxes, K = T or G) adjacent to the DUE-R region (Fig. 1A), which resemble the DnaA-trios essential for DNA replication initiation in *B. subtilis* (Richardson et al. 2016). It remains unknown whether deletion of these putative trio sequences in *E. coli* has any effect on *oriC* activity or initiation timing. *In vitro* evolution of *oriC* revealed that the accumulation of mutations within the DUE (increasing the dA + dT content), together with the introduction of a specific A/T-rich sequence in DUE-M and an increased number of KAK-boxes in DUE-R, facilitate DUE unwinding compared with wt *oriC* (Suzuki and Su’etsugu 2025). It will be of significant interest to determine whether the continuous KAK motifs, the increased dA + dT content, or both are responsible for stabilizing the unwound region at the DUE. Notably, the sharp IHF-mediated DNA bend may eliminate the requirement for trio sites.

Once DnaA·ATP has bound both dsDNA and ssDNA, the melted DNA creates a bubble to which the SSB protein may bind, protecting the ssDNA from degradation and preventing it from refolding (Ozaki et al. 2008, Sakiyama et al. 2017). DnaA·ATP, at the DUE-L and -R sub-motifs, interacts with and loads the replicative DNA helicase DnaB, as part of the DnaB-DnaC complex (Sakiyama et al. 2017, Tsuruda et al. 2026). DnaB is the ubiquitous replicative helicase with a RecA-like fold and belongs to superfamily (SF) 4 of helicases, which are hexameric ring- or spiral-shaped enzymes with 5'→3' polarity (San Martin et al. 1995, Barcena et al. 1998, Bailey et al. 2007). DnaB acts as a recruiting hub, as it physically interacts with DnaA, DnaC, DnaG, the DnaX/τ subunit of the CLC, and the accessory Rep DNA helicase (Schaeffer et al. 2005, Atkinson et al. 2011). The *dnaC* gene is less conserved (Weigel and Seitz 2006), it was likely acquired by horizontal gene transfer from a bacteriophage replicon, and its product, DnaC, is an AAA^+^ ATPase (unrelated to *B. subtilis* DnaC replicative helicase) that forms a dodecameric complex with DnaB [DnaB_6_DnaC_6_ (Barcena et al. 2001)]. DnaC·ATP inhibits DnaB·ATP activity and facilitates its delivery to *oriC* by binding to ssDNA (Davey et al. 2002). The DnaC loader opens the DnaB hexameric ring and stabilizes the open spiral conformation of the complex, facilitating its loading at the unwound DUE region (Arias-Palomo et al. 2013, Nagata et al. 2020). ATP hydrolysis by DnaC is not required to load DnaB onto ssDNA (Arias-Palomo et al. 2013), however, DnaC mutants defective in ATP binding fail to deliver DnaB to *oriC in vitro* and *in vivo* (Davey et al. 2002). Recent studies show that, upon DNA binding, ATP hydrolysis by DnaC leads to closure of the hexameric DnaB·ATP ring around the ssDNA, which subsequently serves as the future lagging-strand template (Arias-Palomo et al. 2019).

For bidirectional replication to occur, two DnaB helicases must be loaded at *oriC*. Functionally distinct DnaA subcomplexes have been observed at the DOR. In addition to the DnaB-loading activity of the DnaA subcomplex at the left half of the DOR, the DnaA subcomplex at the right half of the DOR stimulates DnaB binding/loading (Ozaki and Katayama 2012, Tsuruda et al. 2026). This activity may result in the loading of one DnaBC complex onto the bottom strand and a second complex onto the top strand (Davey et al. 2002).

In certain mutant backgrounds, non-canonical loading of the replicative DNA helicase is observed. If DnaB helicase loading at *oriC* is defective or the interaction between DnaA and DnaB is impaired, the pre-primosomal protein PriC, which is not part of the replisome, assists loading of the DnaBC complex at the unwound *oriC* region (Yoshida et al. 2025). In the absence of *oriC* or in the presence of defective DnaA, inactivation of *rnhA* (encoding RnhA, *a.k.a*. RNase HI) is required for constitutive stable DNA replication (cSDR) (Kogoma and von Meyenburg 1983). In this context, loading of the DnaBC complex occurs outside *oriC* and is catalyzed by the ubiquitous pre-primosomal protein PriA bound to stable R-loops, which form when nascent RNA transcripts pair with the template DNA strand, leaving the non-template strand unpaired. Certain long-living R-loops, together with distinct recombination proteins, provide a primer for leading-strand synthesis and promote cSDR (Kogoma 1997). Moreover, activation of the SOS response induces a second type of SDR (iSDR), which is proposed to initiate from D-loops, which are early intermediates in homologous recombination (Kogoma 1997).

Once the two DnaBC complexes are assembled at *oriC*, displacement of the DnaC loader is required for DnaB activation and further replisome assembly. SSB and the N-terminal domain of DnaB act as hubs to recruit the DnaG primase to the DUE region, forming the DnaB-DnaG complex, termed the primosome (Gao and McHenry 2001, Bailey et al. 2007, Makowska-Grzyska and Kaguni 2010). The DnaB-DnaG complex may adopt variable stoichiometries (DnaB_6_-DnaG_3_ in solution and potentially DnaB_6_-DnaG_1_ in the elongating replisome) (Mitkova et al. 2003, Bailey et al. 2007, Luo et al. 2025). DnaB and SSB appear unable to bind simultaneously DnaG (Bonde et al. 2024). The interaction of DnaB, as part of the DnaBC complex, with DnaG is weak and transient and is regulated by DnaC (Felczak et al. 2017). This interaction is essential for replication initiation, as it favors allosteric dissociation of DnaC, stimulates DnaG-mediated synthesis of a short (∼10-nt long) RNA primer initiated at specific triplet sequences, and activates the DnaB helicase (Johnson et al. 2000, Makowska-Grzyska and Kaguni 2010). The first primer synthesized on each strand at *oriC* becomes the leading-strand primer for the opposing RF, and subsequent primers initiate Okazaki fragment synthesis on the lagging strand.

DnaB alone cannot overcome the block imposed by the DnaA oligomeric complex, whereas DnaB in the presence of SSB can release DnaA molecules from *oriC* (Akama et al. 2025). DnaG induces a conformational change in the N-terminus of DnaB, which enhances the interaction of DnaB with the DnaX/τ subunit of the CLC (Monachino et al. 2020). The CLC is composed of the DnaXZ/γ, DnaX/τ, HolA/δ, HolB/δ’, HolC/χ, and HolD/ψ subunits, with DnaXZ/γ and DnaX/τ subunits both encoded by the *dnaX* gene; DnaXZ/γ is a truncated version of DnaX/τ produced by a ribosomal frameshift (Flower and McHenry 1990). HolC/χ and HolD/ψ, upon interacting with SSB and binding to DnaXZ/γ and DnaX/τ, increase their affinity for HolA/δ and HolB/δ’ (Table 1) (Gao and McHenry 2001). This connection with SSB is essential, as depletion of HolC/χ- HolD/ψ impairs SSB-Pol III interaction and leads to Pol II disassembly and genome instability (Rascon Perez et al. 2025).

DnaX/τ associates with the C-family polymerizing subunit DnaE/α (*a.k.a*. DnaE1) of the Pol III core enzyme (Gao and McHenry 2001, McHenry 2011, Timinskas et al. 2026). The Pol III core enzyme is additionally composed of the proofreading subunit DnaQ/ε, which harbors a DEDDh-family 3'→5' exonuclease domain, and the accessory HolE/θ subunit, which stabilizes DnaQ/ε (McHenry 2011, Monachino et al. 2020). The CLC then remodels SSB on DNA and efficiently loads the ring-shaped DnaN/β sliding-clamp onto ssDNA *via* an opening-and-closing mechanism, with the DnaX/τ subunit bridging the leading- and lagging-strand Pol III holoenzymes (HEs), which consist of the Pol III core enzyme, the CLC, and the DnaN/β sliding-clamp (McHenry 2011). Interaction of DnaX/τ with DnaB facilitates DnaG-mediated primer formation and stabilizes the primosome-Pol III HE interaction (Monachino et al. 2020).

The DnaN/β sliding-clamp interacts with numerous partners through the clamp-binding motif (CBM), although some proteins make additional contacts outside this hydrophobic cleft. DnaE/α interacts with DnaN/β *via* the CBM, increasing the processivity and rate of DNA synthesis by the Pol III HE (Dohrmann and McHenry 2005, Jergic et al. 2013). Finally, DnaB-driven unwinding triggers the transition from initiation to elongation of DNA replication (O’Donnell 2006).

### Replication initiation in *B. subtilis*

DnaA·ATP initially binds to high‐affinity DnaA boxes at the *oriC*1 and *oriC* regions (Fig. 1B). DNA replication initiates when DnaA·ATP also occupies low-affinity sites within *oriC1* and *oriC*, promoting the formation of a loop through interactions between these two neighboring *oriC* regions (Fukuoka et al. 1990, Krause et al. 1997) (Fig. 1B). The upstream *oriC*1 region may serve as replication enhancer for the replication start that occurs at *oriC* (Krause et al. 1997). The *oriC* region additionally contains the DUE, the trinucleotide DnaA target sequences (DnaA-trios), and the DnaD recognition element (DRE) motifs (Fig. 1B) (Yoshikawa and Wake 1993, Richardson et al. 2016, Winterhalter et al. 2023). DnaA·ATP oligomerization is stimulated by *oriC*, ssDNA, or dsDNA, and the extent of oligomerization correlates with the frequency of replication initiation (Scholefield et al. 2012).

DnaA·ATP requires the essential sequence-independent nucleoid-associated proteins Hbsu (encoded by *hbs*) and DnaD to melt the DUE region (Zhang et al. 2005, Karaboja and Wang 2022). The heterologous *E. coli* DnaA protein can form a loop structure between *oriC*1 and *oriC*, but *oriC* melting is species-specific, and only homologous DnaA·ATP can unwind the AT-rich DUE region of *oriC* (Fig. 1B) (Krause et al. 1997). The unwound DUE exposes the essential ssDNA DnaA-trio (*a.k.a*. NAN) binding motifs, on which DnaA·ATP assembles into an oligomer to stabilize the unwound DUE region (Richardson et al. 2016, Pelliciari et al. 2021, 2023). In fact, DnaA·ATP subunits that bind at the junction between the last duplex DnaA box and the first ssDNA trio motif stabilize oligomerized DnaA·ATP on the same strand, thereby promoting strand separation at the DUE (Richardson et al. 2016). Subsequently, to protect ssDNA from degradation and regulate protein access to this region, the SsbA protein may bind to the displaced strands (Jameson and Wilkinson 2017).

A sequential two-stage strategy involving the essential proteins DnaD, DnaB (unrelated to *E. coli* DnaB), and DnaI promotes assembly of the replicative DnaC DNA helicase at the melted DUE region (Velten et al. 2003). The *dnaB, dnaD*, and *dnaI* genes, which are absent in *E. coli*, resemble genes found in phage replicons, and were probably acquired from bacteriophages (Weigel and Seitz 2006). The DnaI AAA^+^ ATPase is the functional counterpart of the *E. coli* DnaC loader, because it forms a complex with DnaC (Table 1). The DnaA initiator plays an indirect role in DnaC recruitment. In fact, the DnaC helicase physically interacts with different protein partners (DnaI, DnaG, and DnaX), but not with DnaA, and DnaI, whose AAA^+^ ATPase domain shares homology with the equivalent domain of DnaA, does not interact with DnaA (reviewed in Jameson and Wilkinson 2017, Blaine et al. 2023).

DnaA interacts with DnaD, which binds specifically to the DRE motifs located opposite to DnaA-trios, facilitating strand opening at *oriC* (Fig. 1B) (Ishigo-Oka et al. 2001). DnaD, in turn, interacts with and recruits DnaB, establishing an ordered pathway of association at *oriC* (Rokop et al. 2004, Bruand et al. 2005, Smits et al. 2010). DnaB also interacts with DnaI, and both proteins interact with DnaC (Velten et al. 2003). DnaI consists of two structured domains. The larger C-terminal AAA^+^ domain contains the nucleotide-binding site and a cryptic ssDNA binding site, whereas the N-terminal domain binds zinc and is essential for interaction with DnaC (Ioannou et al. 2006, Loscha et al. 2009, Tsai et al. 2009). The DnaC helicase is a ubiquitous SF4 helicase with 5’ → 3’ polarity with a RecA-like fold in its C-terminal domain, whereas its N-terminal domain, which is essential for polarity, binds the DnaG primase (Mesa et al. 2006).

In the *Bacillus* genus *(Geobacillus stearothermophilus* and *G. klaustophilus*), DnaC (*a.k.a*. DnaB), as well as the virally encoded G40P, are *bona fide* hexameric DNA helicases (Barcena et al. 1998, Ioannou et al. 2006, Lo et al. 2009). In contrast, the *B. subtilis* DnaC helicase is less stable and appears to form a mixture of oligomeric states, with the hexameric closed ring form being inactive in unwinding (Velten et al. 2003). Notably, *G. stearothermophilus* and *G. klaustophilus* DnaC helicases form a stable complex with DnaI, with a DnaI_6_-DnaC_6_ stoichiometry that is loaded onto ssDNA in the presence of ATP (Tsai et al. 2009, Liu et al. 2013), suggesting that these helicases are loaded onto both strands of *oriC via* the ring-breaking mechanism. Thus, within the *Bacillus* Genus, either an unstable DnaC·ATP oligomer or a heterododecameric, ring- or spiral-shaped DnaI·ATP-DnaC·ATP complex is assembled at *oriC* using ring-making or ring-breaking mechanisms, respectively (Velten et al. 2003, Tsai et al. 2009, Liu et al. 2013).

The precise molecular mechanisms by which the DRE-DnaD-DnaB or (DnaA)-DnaD-DnaB complexes regulate the timing, location, and orientation of the two DnaI-DnaC complexes on both *oriC* strands remain poorly understood. The DnaD-DnaB pair may control replicative helicase loading. DnaB and DnaD assemble as oligomers and bind both ssDNA and dsDNA (Marsin et al. 2001, Bruand et al. 2005). They have been described as DNA-remodeling proteins with opposing activities: DnaD condenses, whereas DnaB relaxes, supercoiled DNA (Zhang et al. 2005). In addition, DnaD stimulates DnaB binding to ssDNA (Marsin et al. 2001). It has been proposed that replication initiation is controlled, at least in part, by regulating the interaction between DnaD and the membrane-associated protein DnaB (Rokop et al. 2004).

Whether replication can initiate in the absence of DnaA and/or *oriC via* PriA-mediated loading of the replicative DnaC helicase at stable R-loops remains unknown. Rescue of the thermosensitive phenotype in Δ*rnhC dnaA*1*ts* cells has not been observed in *B. subtilis* (Moriya et al. 1990, B.C. unpublished results), in contrast to *E. coli*, where mutants lacking DnaA or *oriC* and RnhA [*a.k.a*. RNase HI, functional counterpart of *B. subtilis* RnhC (*a.k.a*. RNase HIII)] can overcome lethality by initiating cSDR at R-loops (Kogoma 1997). Can the replicative helicase be loaded at *oriC* in the absence of any of these essential players? A DnaB S371P mutation suppresses the requirement for DnaD (Rokop et al. 2004, Bruand et al. 2005), but the precise mechanism by which DnaB S371P, together with DnaI, promotes DnaC loading at *oriC* remains elusive. There is no evidence for a direct interaction between DnaA and DnaB, which binds DNA with a preference for fork-like substrates (Marsin et al. 2001). Notably, *dnaB* S137P cells are more sensitive to *dnaA* overexpression than *dnaB*^+^ cells (Rokop et al. 2004). The three-dimensional structure of DnaB exhibits an overall architecture similar to that of the *E. coli* CLC, which loads the ring-shaped processivity factor DnaN *via* a ring-breaking mechanism (Núñez-Ramírez et al. 2007), suggesting a potential role for DnaB as a ring breaker and loader. Whether DnaB S371P contributes to opening the ring- or spiral-shaped DnaI·ATP-DnaC·ATP complex remains to be determined.

As part of the DnaI-DnaC complex, DnaC interacts with and recruits the DnaG primase, forming a ternary complex (Bailey et al. 2007, Liu et al. 2013, Rannou et al. 2013). ATP hydrolysis by DnaI may be stimulated upon ssDNA binding, thereby promoting its dissociation from the ssDNA-DnaI-DnaC complex (Ioannou et al. 2006). Other studies showed that DnaI is released when DnaG and the essential error-prone DNAP DnaE (*a.k.a*. DnaE3) interact with DnaC to form a DnaG-DnaC-DnaE ternary complex (Rannou et al. 2013). DnaC activation may be allosterically accelerated by ribonucleotides and by DnaG binding to DnaC (Soultanas 2002, Ioannou et al. 2006, Rannou et al. 2013). This results in formation of an active DnaC-DnaG complex, in which DnaG stimulates the DNA helicase activity of DnaC (Ayora et al. 1998, Bailey et al. 2007, Rannou et al. 2013). In contrast, DnaI inhibits DnaG-mediated stimulation of DnaC helicase activity (Soultanas 2002). It has been proposed that DnaC may stimulate DnaG activity by increasing the local concentration of ssDNA substrate and by ensuring that multiple DnaG subunits are in close proximity (Bailey et al. 2007). The sequence recognized by DnaG at these pre-priming sites remains elusive.

Once released from DnaI, DnaC encircles ssDNA and unwinds forked DNA with 5'→3' polarity (McHenry 2011). DnaC, SsbA, and DnaG interact with and contribute to recruitment of DnaE (Costes et al. 2010, Rannou et al. 2013). DnaE forms a ternary complex with DnaC and DnaG of undefined stoichiometry, referred to as the primosome (Rannou et al. 2013). DnaC additionally interacts with and recruits DnaX, a component of the CLC, which is composed of DnaX, HolA, and HolB (Table 1) (Martínez-Jiménez et al. 2002, Haroniti et al. 2004, Afonso et al. 2013). Within the CLC, DnaX interacts with and recruits both leading- and lagging-strand PolC, a dual-function DNAP enzyme with 3′→5′ exonuclease proofreading and 5'→3' polymerizing domains (Sanjanwala and Ganesan 1991, McHenry 2011). No interaction between DnaE and DnaX has been described (Bruck and O’Donnell 2000). The CLC additionally interacts with and loads two ring-shaped DnaN sliding-clamps onto each complementary strand at *oriC* in an ATP-dependent manner, forming the PolC HEs (Bruck and O’Donnell 2000, Sanders et al. 2010). Both C-family DNAPs, PolC and DnaE, contain canonical CBMs for interaction with DnaN. However, whereas the DnaN-PolC interaction is essential, the DnaN-DnaE interaction is dispensable for DNA replication (O’Neal et al. 2025). DnaX bridges the leading- and lagging-strand PolC HEs through protein-protein interactions (McHenry 2011). Notably, DnaE is part of the *B. subtilis* SsbA C-terminal interactome, but neither PolC nor other replisome components depend on the SsbA C-terminal domain for targeting to active chromosomal RFs (Costes et al. 2010).

Finally, the DnaC helicase unwinds dsDNA and generates ssDNA that is used by the primase-polymerase DnaG-DnaE complex to synthesize the leading-strand hybrid primer *in trans* (as a functional counterpart of the eukaryotic Polα-primase complex), with DnaE handing off the hybrid primer to the replicative PolC core enzyme (Seco and Ayora 2017). During lagging-strand priming, the primosome complex (DnaC-DnaG-DnaE) enables DnaC-mediated unwinding and facilitates synthesis of the lagging-strand hybrid primer by DnaG-DnaE, which is subsequently handed off to PolC core enzyme (Sanders et al. 2010). DnaC-driven unwinding triggers the transition from initiation to elongation of DNA replication (McHenry 2011).

### Replication elongation

Replication elongation of genomic DNA is broadly conserved in both *E. coli* and *B. subtilis*, with some differences. Bidirectional chromosomal replication is carried out by two replisomes. *Bacillus subtilis* displays a relatively stationary factory, with an apparent single replisome focus that reversibly splits into two closely spaced foci every few seconds during replication (Lemon and Grossman 1998, Berkmen and Grossman 2006, Mangiameli et al. 2017a). These foci are positioned either at midcell (in cells with a single nucleoid) or symmetrically within each half of the cell (in cells with two nucleoids), with both replisomes remaining close to each other during ∼80% of the replication cycle (Mangiameli et al. 2017a). In *E. coli*, time-lapse analysis of slowly growing cells, showed that the left and right replichores track independently around the chromosome (reviewed in Reyes-Lamothe et al. 2012). However, recent studies in other growth conditions have shown that the *E. coli* chromosome moves to the replisome (Gras et al. 2024) and that bidirectionally moving replisomes are bound to each other during the first half of the replication period, and free from each other during the second half (Chen et al. 2023).

At the head of the RF at each replisome is the primosome (composed of two proteins in *E. coli* and three in *B. subtilis*). DNA synthesis is carried out by two single replicases, one responsible for leading-strand synthesis and the other for lagging-strand synthesis. The precise mechanism by which these two replicases are coordinated is not fully understood. The DNAP HEs of both model bacteria are tripartite (a DNAP core, a sliding-clamp, and the CLC) and require a primer to initiate DNA synthesis (O’Donnell 2006, McHenry 2011). However, these DNAPs are significantly divergent. Indeed, the polymerizing DnaE/α subunit of the *E. coli* replicative Pol III core enzyme shares < 20% sequence identity with the replicative PolC core enzyme of *B. subtilis*. The *E. coli* Pol III HE, which is a complex of 10 distinct proteins (see Table 1), extends short RNA primers synthesized by DnaG (reviewed in McHenry 2003, O’Donnell 2006, Lazowski et al. 2024). In contrast, *B. subtilis* PolC HE, which is a complex of 5 different proteins (see Table 1), only extends 3'-OH dNMPs, i.e. RNA-DNA hybrid primers, but not RNA primers (Sanders et al. 2010, Seco and Ayora 2017). In both model bacteria, the replicative DNA helicase unwinds dsDNA at a low rate (<50 nucleotide (nt) s^–1^) *in vitro* (Kim et al. 1996, Sanders et al. 2010), but unwinding is greatly stimulated by replisome subunits to support the *in vivo* rates of replisome progression (≥500 nt s^–1^) (McHenry 2011). For replication elongation to proceed on circular chromosomes, the activity of topoisomerases is essential (Chen et al. 2013, Kim and Guo 2024).

### Replication elongation in *E. coli*

**Figure 2:**
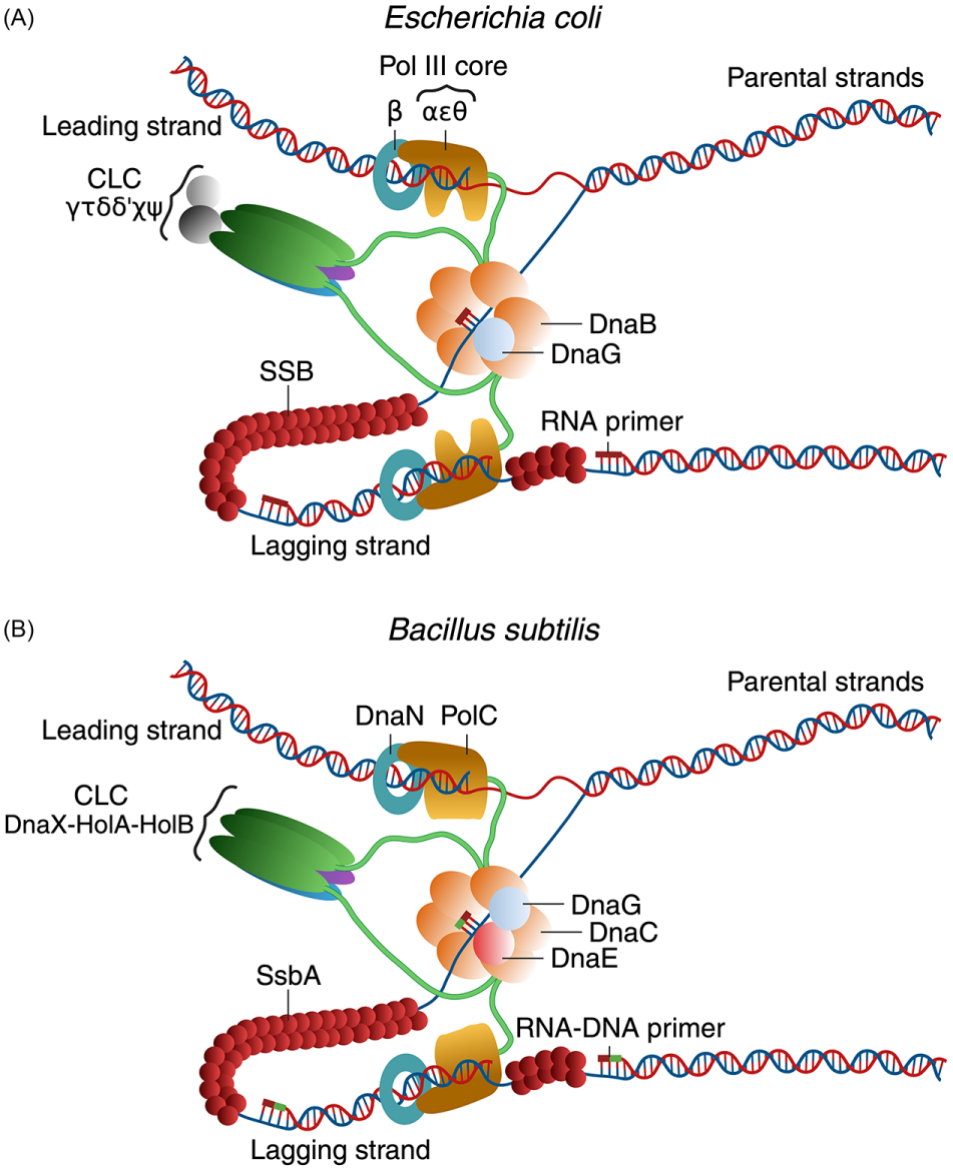
Schematic representation of the replisome complex in *E. coli* (A) and *B. subtilis* (B). (A) The hexameric DnaB helicase interacts with the DnaG primase to form the primosome. The DnaB–DnaG complex unwinds duplex DNA and synthesizes the RNA primer. DnaB also interacts with the DnaX/τ subunit of the clamp loader complex (CLC). The CLC (DnaXZ/γ, DnaX/τ, HolA/δ, HolB/δ’, HolC/χ, and HolD/ψ) anchors the leading- and lagging-strand replicases to the helicase through interaction with DnaE/α, and also recruits and loads the DnaN/β sliding-clamp. The Pol III core enzyme (DnaE/α-DnaQ/ε-HolE/θ) interacts with DnaN/β and with DnaX/τ *via* the DnaE/α subunit to form the Pol III holoenzyme (HE). The lagging-strand template ssDNA is coated by SSB, which, together with the Pol III HE and the primosome, constitutes the replisome. (B) The hexameric DnaC helicase interacts with DnaG, and DnaG interacts with DnaE to form the primosome. The DnaC–DnaG–DnaE primosome complex unwinds the DNA substrate and synthesizes a hybrid RNA–DNA primer. The CLC, composed of only three subunits (DnaX, HolA, and HolB), loads the DnaN sliding-clamp. DnaC interacts with the DnaX subunit of the CLC. The CLC anchors the leading- and lagging-strand replicases to the helicase through interaction with PolC, which also interacts with the DnaN sliding-clamp. The PolC core enzyme, together with DnaN and the CLC, forms the PolC HE. The lagging-strand template ssDNA is coated by SsbA, which, together with the PolC HE and the primosome, constitutes the replisome. (A, B) Replisome cartoons are not drawn to scale. For clarity, interactions between SSB/SsbA and other replisome components are not depicted.

Each replisome, which is divergently recruited at *oriC*, is organized into three discrete subassemblies consisting of the leading- and lagging-strand replicative Pol III HEs, the primosome (DnaB and DnaG), and the SSB protein (Fig. 2A) (O’Donnell 2006, McHenry 2011). Accessory factors may also contribute to the elongation process. Indeed, the dispensable SF1A 3'→5' DNA helicase Rep, which interacts with the DnaB helicase, colocalizes with 70% of RFs (Syeda et al. 2019).

A reconstituted replisome lacking the dispensable DnaXZ/γ subunit of the CLC contains three Pol III core enzymes, whereas a fully reconstituted wt replisome contains only two Pol III core enzymes (McInerney et al. 2007, Dohrmann et al. 2016). Since a replisome mutant in the DnaXZ/γ subunit impairs survival after UV treatment, reduces Pol IV (*a.k.a*. DinB)-mediated mutagenesis, and affects F plasmid maintenance under physiological conditions, it has been inferred that the DnaXZ/γ subunit is an intrinsic part of the replisome (Dohrmann et al. 2016); thus, the role of the replisome architecture involving three Pol III core enzymes remains elusive.

The Pol III HEs, which are high-speed and high-fidelity replicative DNAPs, carry out the bulk of DNA synthesis (O’Donnell 2006, McHenry 2011). The leading strand is replicated in a nearly continuous manner, while the lagging strand is synthesized discontinuously in 1000-2000 nt-long stretches known as Okazaki fragments (Ogawa and Okazaki 1980). The initiation of each Okazaki fragment requires priming by DnaG, with the CLC potentially recognizing the primer-template junction through the interaction of the HolD/ψ-HolC/χ complex with SSB, and loading the DnaN/β sliding-clamp at this site (Glover and McHenry 1998). Through cycles of DNA extension following primer synthesis, Pol III hops from the original DnaN/β to a newly loaded clamp, resulting in discontinuous DNA synthesis (Ogawa and Okazaki 1980, Stukenberg et al. 1994). DnaB helicase remains stably bound to the lagging-strand template (Kornberg and Baker 2005, Beattie et al. 2017). DnaG must dissociate from DnaB to be recycled. The three-point switch model proposes that after RNA primer synthesis, DnaG remains bound to the primer through interaction with SSB (Yuzhakov et al. 1999). The interaction of the HolC/χ subunit of the CLC with SSB displaces DnaG from the SSB-DnaG complex, facilitating access of the Pol III HE to the nascent primer and DnaG recycling (Yuzhakov et al. 1999). The DnaN/β sliding-clamp molecules left on DNA must be actively unloaded and recycled, because their cellular content is >5-fold lower than the number of Okazaki fragments required for one round of chromosomal replication, and live-cell studies revealed a stoichiometry of about two DnaN/β sliding-clamps per RF (Leu et al. 2000, Reyes-Lamothe et al. 2010).

During DNA synthesis, the DnaE/α DNAP subunit polymerizes DNA and discriminates between correct and incorrect incoming nucleotides, while the DnaQ/ε subunit removes misincorporated nucleotides, whereas the HolE/θ subunit lacks enzymatic activity. The Pol III HE sequentially displaces SSB from the lagging-strand template, with the DnaN/β sliding-clamp increasing replicase processivity upon interaction with the DnaE/α and DnaQ/ε subunits of the Pol III core enzyme (Dohrmann and McHenry 2005, Jergic et al. 2013). At the lagging strand, the presence of a new primer, rather than primer synthesis *per se* or collision of an elongating DNAP with the 5'-end of the preceding Okazaki fragment, triggers Pol III core enzyme cycling (Yuan and McHenry 2014).

Okazaki fragment maturation involves highly coordinated reactions that remove RNA primers and convert the discrete fragments into a continuous lagging strand. Upon Pol III disassembly, the 5'-end of the preceding Okazaki fragment generates a flap (Okazaki et al. 1971, Kornberg and Baker 2005). The A-family DNAP Pol I (*a.k.a*. PolA), which consists of a flap endonuclease/5'→3'-exonuclease at the N-terminus, a 3'→5' exonuclease with proofreading activity in the central region, and a 5'→3' polymerase domain at the C-terminus, removes the RNA primer of the downstream Okazaki fragment *via* its flap endonuclease and 5'→3'-exonuclease activities (Okazaki et al. 1971, Ogawa et al. 1984, Botto et al. 2023). A Pol I mutant lacking only the flap endonuclease/5'→3'-exonuclease nuclease activity is not viable, and viability can be restored by providing the endonuclease/5'→3'-exonuclease domain *in trans* (Joyce and Grindley 1984). Primer removal can also be partly performed by the RnhA nuclease (Okazaki et al. 1971, Ogawa et al. 1984).

The SSB protein, which is involved in multiple aspects of DNA metabolism, including replication, repair, and recombination, is an essential component of each replisome (reviewed in Bonde et al. 2024, and references therein). The C-terminal tails of tetrameric SSB are the primary sites for organizing replisome transactions, with an *ssb* variant lacking the last 8 codons (*ssb*ΔC8) being lethal, and a mutation affecting the penultimate residue (the *ssb*113 mutation, SSB P176S) conferring a thermosensitive phenotype (reviewed in Bonde et al. 2024). SSB performs three distinct functions: (i) it binds lagging-strand template ssDNA with high affinity, preventing the formation of secondary structures, protecting ssDNA from degradation, and avoiding self-annealing during DnaB-mediated unwinding (Bonde et al. 2024); (ii) it stimulates strand displacement during DNA synthesis (Yuan and McHenry 2009); and (iii) it serves as a hub for the assembly of multiple replication proteins, including the HolC/χ subunit of the Pol III HE, B-family Pol II (*a.k.a*. PolB), Y-family Pol IV (*a.k.a*. DinB) and Pol V (*a.k.a*. UmuCD'_2_), DnaG, PriA, PriB, PriC, Topo III (*a.k.a*. TopBα), and repair proteins such as RecO, RecG, RecJ, RecQ, DinG, RadD, and RarA (Bonde et al. 2024). DNA repair proteins and translesion synthesis (TLS) DNAPs may not travel with RFs in the absence of environmental stress, as documented for PriA (Rascon Perez et al. 2025), suggesting that while such protein-protein interactions are necessary for overcoming replication stress, they are not sufficient to constitutively link a repair toolbox to an active RF.

Ancillary SF1A DNA helicases, such as Rep (or UvrD in the Δ*rep* context), help RFs to progress through hard-to-replicate regions, and absence of accessory DNA helicases results in a synthetic lethal phenotype (Guy et al. 2009). Rep and UvrD may dislodge protein barriers, and UvrD also alleviates replication-transcription conflicts (RTCs).

### Replication elongation requires two C-family DNAPs in *B. subtilis*

Two replisomes assembled at *oriC* initiate bidirectional DNA replication (Murray et al. 2017). Each replisome consists of three discrete subassemblies: the leading- and lagging-strand PolC HEs, the primosome proteins (DnaC, DnaG, and DnaE) bridged *via* the DnaX subunit of the CLC, and the SsbA protein (Fig. 2B) (Bruck and O’Donnell 2000, Le Chatelier et al. 2004, Rannou et al. 2013, Paschalis et al. 2017). The PolC HE is composed of five subunits [the PolC core, the CLC (DnaX-HolA-HolB), and the DnaN sliding-clamp] (Table 1) (Bruck and O’Donnell 2000, Sanders et al. 2010). The contribution of an accessory SF1A DNA helicase remains poorly understood.

Genome replication requires two essential C-family DNAPs, PolC and DnaE (Dervyn et al. 2001, Timinskas et al. 2014). Neither PolC nor DnaE can initiate *de novo* DNA synthesis. PolC can only extend 3'-OH dNMP ends (Sanders et al. 2010, Seco and Ayora 2017), whereas DnaE can extend 3'-OH rNMP and 3'-OH dNMP ends (Sanders et al. 2010). PolC is a bifunctional enzyme that contains a 5'→ 3' polymerase domain and a DEDDh-family 3'→ 5' proofreading exonuclease domain within a single polypeptide (Bruck and O’Donnell 2000). The PolC proofreading domain efficiently corrects errors introduced by the polymerase domain, ensuring high replication fidelity, although the mechanistic details of the transition from polymerization to proofreading remain poorly understood. *In vitro* reconstitution assays revealed that the PolC core enzyme, which requires RNA-DNA hybrids to prime DNA synthesis, functions as a processive, high-speed DNAP (Sanders et al. 2010, Seco et al. 2013, Seco and Ayora 2017). In contrast, DnaE is a low-speed, error-prone, distributive DNAP lacking proofreading activity and was therefore considered a TLS DNAP (Bruck and O’Donnell 2000, Bruck et al. 2003, Le Chatelier et al. 2004, McHenry 2011). As already mentioned, DnaE forms the ternary primosome complex with DnaC and DnaG, and assembles at *oriC* prior to PolC (Rannou et al. 2013).

Early experiments suggested that DnaE was the lagging-strand DNAP, because upon DnaE depletion only leading-strand synthesis of a σ-type (*a.k.a*. rolling circle-type) replicating plasmid was observed. (Dervyn et al. 2001). This hypothesis was partially supported by using an *in vitro* reconstituted replication system using a σ-type replication substrate that mimics a stalled fork in which the leading strand contains a 3'-OH dNMP end (Sanders et al. 2010). In this system, PolC can extend the leading strand at a physiologically relevant elongation rate (∼500 nt s^−1^) in the absence of DnaG and DnaE (Sanders et al. 2010, Seco et al. 2013), whereas lagging-strand synthesis requires *de novo* priming by the primosome (DnaG-DnaE-DnaC). In addition, DnaE activity is too slow (∼25 nt s^−1^) to keep pace with the speed of the RF (Sanders et al. 2010, Seco et al. 2013). In such *in vitro* reconstituted assays, leading-strand DNA synthesis with DnaE as the sole DNAP is inhibited by DnaG in the presence of SsbA, whereas SsbA stimulates PolC-mediated leading-strand DNA synthesis (Seco and Ayora 2017). All these *in vitro* experiments showed that DnaE adds few dNMPs to the DnaG-synthesized RNA pre-primer to form a hybrid RNA-DNA primer in a manner analogous to the eukaryotic Polα-primase complex (reviewed in Pellegrini 2012, and references therein). Then, DnaE hands off this hybrid RNA-DNA primer to the PolC HE for leading- and lagging-strand DNA synthesis (Sanders et al. 2010, Seco et al. 2013). *In vitro* reconstitution experiments using a *bona fide* θ-type replication substrate, in which *de novo* priming is required for both leading- and lagging-strand DNA synthesis, showed that replication initiation does not occur when DnaE was omitted (Seco and Ayora 2017). These discoveries clearly revealed a role for DnaE also in primer synthesis for leading-strand replication. Wherever *de novo* priming is required, DnaE adds a few dNTPs complementary to the template strand to the DnaG-synthesized RNA primer, generating RNA-DNA hybrid primers that are handed off to the PolC HE, which performs the bulk of DNA synthesis (Seco and Ayora 2017). In parallel, the CLC recruits DnaN to the primer-template junction (Su’etsugu and Errington 2011), but the interaction of DnaN with DnaE is dispensable (O’Neal et al. 2025). After Okazaki fragment synthesis, PolC assembles at the next hybrid primer where DnaN has been loaded, leaving the previous DnaN behind. ∼200 DnaN sliding-clamps accumulate behind the RF in so called “clamp zones” (Su’etsugu and Errington 2011), which have been proposed to recruit proteins bearing a CBM (e.g. mismatch repair proteins, etc.) (Lenhart et al. 2016).

Okazaki fragment maturation involves several non-essential nucleases in a hierarchical manner: FenA (*a.k.a*. YpcP or ExoR), the A-family DNAP PolA (*a.k.a*. Pol I), and RnhC. Genetic analyses reveal that a Δ*fenA* mutation is synthetically lethal in a Δ*polA* background (Fukushima et al. 2007), whereas Δ*rnhC* Δ*polA*, Δ*rnhC* Δ*fenA*, and Δ*rnhB* Δ*rnhC* Δ*polA* strains are temperature sensitive but viable at 37 °C; moreover, *rnhC* is not epistatic with either *polA* or *fenA* (Lowder and Simmons 2023). FenA (absent in *E. coli*) is a standalone flap endonuclease/5'→3'-exonuclease (Randall et al. 2019, Lowder and Simmons 2023). PolA contains a flap endo/5'→3'-exonuclease domain at the N-terminus, and a 5'→3' polymerase domain at the C-terminus, but lacks a 3’→5’ proofreading domain (Kiefer et al. 1997). RnhC is a ribonuclease that degrades RNA-DNA hybrids embedded in DNA, with or without a covalent RNA-DNA junction (Ohtani et al. 1999, Randall et al. 2019). Current knowledge indicates that, in *B. subtilis*, Okazaki fragment maturation can proceed *via* two pathways. In the primary pathway, FenA removes RNA flaps. In the secondary pathway, RNase HIII cleaves the RNA primer internally, shortening the RNA-DNA hybrid to facilitate PolA nuclease activity (Randall et al. 2019, Lowder and Simmons 2023). After primer removal, the DNAP responsible for gap filling remains poorly defined, as PolA is a mutagenic enzyme (Duigou et al. 2005). We propose that PolC -the only DNAP with proofreading activity- fills the resulting gap.

SsbA, as part of each replisome, has three distinct activities: (i) binding lagging-strand template ssDNA with high affinity to prevent self-annealing of nascent strands and the formation of secondary structures; (ii) protecting ssDNA from degradation and regulating protein access to the ssDNA region; and (iii) serving as a crucial platform for recruiting replication proteins such as DnaE and PriA, primarily through its C-terminal domain, and DNA repair proteins such as RecD2, RecG, RecJ, RecO, RecQ, RecS, and RarA to elongating and/or stalled replisomes (reviewed in Carrasco et al. 2024). It has been hypothesized that at least DnaE, PriA, and RecO colocalize with and travel with replisomes in unperturbed cells *via* their interaction with SsbA (Lecointe et al. 2007, Costes et al. 2010). An *ssbA* mutant variant lacking the last 35 codons (*ssbA*ΔC35) is viable but exhibits a thermosensitive phenotype (Costes et al. 2010). Unexpectedly, the thermosensitive phenotype of *ssbA*ΔC35 cells is suppressed by ectopic RecO overexpression, but not by overexpression of the essential PriA or DnaE proteins (Costes et al. 2010).

Several helicases (RecQ, RecS, RecD2, and RecG), which interact with SsbA, have been proposed to function as accessory DNA helicases during replication elongation (Lecointe et al. 2007, Costes et al. 2010). A combination of Δ*recG* and Δ*recD2* mutations is lethal (Torres et al. 2017), but little information is available about the need for an accessory DNA helicase during replication elongation. The essential SF1A DNA helicase PcrA (the functional homologue of *E. coli* UvrD) physically interacts with RNAP and RecA (Costes et al. 2010, Carrasco et al. 2025), but shows no obvious interaction with the replisome (Carrasco et al. 2024). PcrA alleviates RTCs, prevents RecA from promoting unnecessary recombination during RF repair *in vivo*, and dislodges RecA from ssDNA *in vitro* (Merrikh et al. 2015, Carrasco et al. 2022).

### Replisome dynamics and stability in *E. coli*

The choreography of leading- and lagging-strand synthesis is loosely coordinated, but the leading-strand Pol III core enzyme is physically linked to DnaB through DnaX/τ interactions (Graham et al. 2017). The replicative Pol III* (Pol III HE lacking DnaN/β) is replaced every few seconds in unperturbed cells (Beattie et al. 2017, Lewis et al. 2017). In contrast, DnaB, which overcomes protein barriers on duplex DNA under physiological conditions, is highly stable at RFs (dwell time >15 min) (Beattie et al. 2017, Spinks et al. 2021).

Are other DNAPs part of the replisome? The *E. coli* genome encodes five DNAP enzymes, three of which have a proofreading domain: Pol I, Pol II, and the Pol III core enzyme (Table 1). The B-family DNAP, Pol II, may serve as a backup replicase for the Pol III HE at terminal mismatches and can extend certain damaged templates, acting as a TLS DNAP (Fijalkowska et al. 2012). In addition, the Y-family DNAPs, Pol IV and Pol V, which lack a proofreading domain, are specialized TLS DNAPs (Goodman and Woodgate 2013, Fujii and Fuchs 2020). Under unperturbed conditions, <0.1% of cells exhibit increased expression of TLS DNAPs, and the cellular levels of Pol II and of the Y-family DNAPs (Pol IV and Pol V) increase following SOS induction (reviewed in Fijalkowska et al. 2012). TLS DNAPs appear to function during replication stress through sequential DNAP switching and at the post-replicative level by filling gaps that arise after Pol III HE stalling and repriming downstream of blocking DNA lesions (reviewed in Goodman and Woodgate 2013, Marians 2018, Fujii and Fuchs 2020, and references therein).

*In vitro* reconstitution studies have shown that, at physiological concentrations, Pol III HE and Pol I are incapable of replicating damaged DNA. All five DNAPs possess one or two canonical CBMs (López de Saro et al. 2003). In unperturbed cells, the DnaE/α and DnaQ/ε subunits of the Pol III core enzyme occupy both clefts of the DnaN/β-clamp dimer, thereby preventing Pol II and both TLS DNAPs from interacting with the DnaN/β sliding-clamp (Chang et al. 2019). Single-molecule analysis revealed that Pol IV does not exchange with Pol III unless a template lesion is encountered and one DnaN/β binding cleft is freed from DnaQ/ε. It is therefore likely that Pol II, Pol IV, and Pol V do not travel with the replisome and that their access to the DnaN/β sliding-clamp is competitively regulated, with the dynamic interaction between the DnaQ/ε and DnaN/β subunits playing a central gatekeeping role (Chang et al. 2019). The SSB protein plays also a key role in this recruitment (Chang et al. 2022). Consistent with this, live-cell studies show that 90% of Pol IV and 95% of Pol V foci localize distal to replisomes and contribute to error-prone DDT at the post-replicative level (Robinson et al. 2015, Henrikus et al. 2018). The mechanisms of post-replicative TLS are beyond the scope of this review, and readers are directed to recent specialized reviews (Goodman and Woodgate 2013, Marians 2018, Fujii and Fuchs 2020). Whether TLS DNAPs can replace the Pol III enzyme at the spontaneously stalled forks remains largely unresolved.

### Replisome dynamics and stability in *B. subtilis*

Live-cell imaging of unperturbed cells has revealed the dynamics of the replicative DNAPs. PolC is continuously replaced by cytosolic PolC every few seconds, but DnaE exhibits no measurable dynamics or dwell time (Liao et al. 2016, Li et al. 2019). These results are consistent with *in vitro* data showing that DnaE primarily contributes to the formation of hybrid RNA-DNA primers rather than to replication elongation. The dwell-time of PolC significantly decreases (i.e. the exchange rate increases) upon encountering bulky or non-bulky lesions, as well as during collisions with roadblocks or arrays of RNAPs transcribing highly expressed genes (reviewed in Merrikh et al. 2012, Torres et al. 2025). A reduction in PolC dwell time (i.e. the exchange rate increases) is observed when cells are treated with 6(p-Hydroxyphenylazo)uracil (HPUra), a specific inhibitor of PolC polymerizing activity (Brown 1970), whereas DnaE remains too dynamic for reliable tracking (Liao et al. 2016, Li et al. 2019). In the presence of HPUra, DnaX foci persist, and two distinct CLC sub-populations emerge: one with a dwell time similar to that of PolC, and another with a significantly longer dwell time (Liao et al. 2016, Li et al. 2019). It is conceivable that the longer-dwelling DnaX fraction represents a fraction of CLCs that, in concert with the DnaN sliding-clamp, are involved in recruiting additional proteins required to overcome replication stress.

*In vivo*, the replisome spontaneously disassembles at leading- or lagging-strand template barriers/lesions. This is consistent with the observed stoichiometries of the replicative helicase DnaC and the PolC HE (i.e. PolC or DnaX), showing that only one active replisome is detected in >40% of unperturbed cells (Mangiameli et al. 2017b). Barriers/lesions that stall RFs are likely circumvented by error-free fork remodeling *via* DDT sub-pathways (fork reversal or template switching). The mechanisms of fork remodeling upon fork stalling are beyond the scope of this review, and readers are directed to recent specialized reviews (Cox et al. 2023, Carrasco et al. 2024, Torres et al. 2025).

Are other DNAPs part of the replisome? *Bacillus subtilis* encodes five DNAPs linked to DNA replication (Lenhart et al. 2012, Carrasco et al. 2024), of which only PolC possesses a proofreading domain. The remaining four DNAPs -DnaE, PolA, and the Y-family PolY1 (*a.k.a*. YqjH) and PolY2 (*a.k.a*. YqjW)- lack proofreading domains. The DnaN sliding-clamp interacts with DNAPs *via* their CBM, being the interaction with PolC essential, and with PolA, PolY1, and PolY2 crucial (Duigou et al. 2004, Duigou et al. 2005, O’Neal et al. 2025). In contrast, the DnaN-DnaE interaction is dispensable, and strengthening this interaction leads to a significant increase in UV-induced mutagenesis (O’Neal et al. 2025). The expression of PolY2, and to a lesser extent of DnaE, is damage-inducible, whereas the other DNAPs are constitutively expressed (Le Chatelier et al. 2004, Au et al. 2005).

PolY1 and PolY2 are proposed counterparts of *E. coli* Pol IV and UmuC, respectively (Duigou et al. 2004). PolY2 has been shown to exhibit DNAP activity without the need for additional accessory factors (Patlan et al. 2018), and inactivation of YqjX -a potential UmuD-like protein- does not significantly affect spontaneous or UV-induced mutagenesis, either in the absence of or upon overproduction of PolY2 (Duigou et al. 2004). However, AlphaFold modelling predicts a Mutasome-like complex composed by three proteins (PolY2-YqjX-RecA), in which the RecA-NT motif of PolY2 interacts with RecA, while the N-terminal arm of YqjX interacts with PolY2 (Timinskas et al. 2026). It therefore remains unclear whether PolY2 functions as a standalone TLS DNAP or operates in concert with RecA and YqjX.

Live-cell imaging reveals that PolY1 and PolA are moderately enriched at or near sites of replication in the absence of DNA damage through interactions with the DnaN sliding-clamp (Marrin et al. 2024), whereas PolA only becomes significantly enriched at RFs upon DNA damage induction (Hinrichs and Graumann 2024). Several studies show that spontaneous mutation rates in *polY1* and *polY2* knockout strains are essentially identical to wt in untreated cells, but the *polY1 polY2* double mutant is blocked in TLS (Duigou et al. 2004, Raguse et al. 2017, Romero et al. 2019), suggesting partial redundancy between these TLS DNAPs. In addition, PolY1 has been implicated in normal replication, possibly in alleviating RTCs, as PolY1 promotes lagging-strand mutagenesis when such conflicts are induced at specific loci (Million-Weaver et al. 2015a). Together, these findings suggest that in *B. subtilis*, if error-free DDT pathways fail to circumvent barriers/lesions, TLS DNAPs should bypass damaged or distorted templates at RFs *via* error-prone DDT sub-pathways (reviewed in Carrasco et al. 2024). Several questions remain unresolved: i) whether the small fraction (∼10%) of PolY2-dependent UV-induced mutagenesis is independent of PolA (Duigou et al. 2005), pointing to a poorly defined PolY2-specific pathway; and ii) whether DnaE competes with *bona fide* TLS DNAPs for lesion bypass. Contradictory results have been reported for DnaE: overexpression of DnaE does not increase the spontaneous mutation rate in wt cells, whereas depletion of DnaE reduces UV-induced mutagenesis (Le Chatelier et al. 2004).

### Replication termination

Bidirectional replication of a circular chromosome concludes when the two replisomes converge in the terminus region, located opposite the *oriC* region. Central features of replication termination are broadly conserved (Dewar and Walter 2017), as the resolution of the topological stress (Chen et al. 2013, Kim and Guo 2024). During replication elongation, (+) supercoils accumulate ahead of the RFs and (-) ones behind them (Hiasa and Marians 1996), as previously observed during transcription (Wu et al. 1988). If excessive supercoiling accumulates, replication ceases. The opposing activities of a type II topoisomerase (Topo II/GyrAB), which introduces (−) and relieves (+) supercoiling ahead of the RF, and a type I topoisomerase (Topo I/TopA), which removes (−) supercoils, maintain supercoiling levels compatible with RF progression (reviewed in Wang 2002). Alternatively, as replication progresses, the entire RF can rotate to counteract overwinding of unreplicated DNA, leading to the formation of precatenanes, which are resolved only by type II topoisomerases (Hiasa and Marians 1996, Chen et al. 2013, Cebrian et al. 2015). When the RFs converge, the replisomes disassemble, gaps are filled, nascent leading and lagging strands are ligated, and the resulting fully replicated chromosomes must be decatenated by Topo IV (ParCE) (Zechiedrich and Cozzarelli 1995, Ullsperger and Cozzarelli 1996).

**Figure 3:**
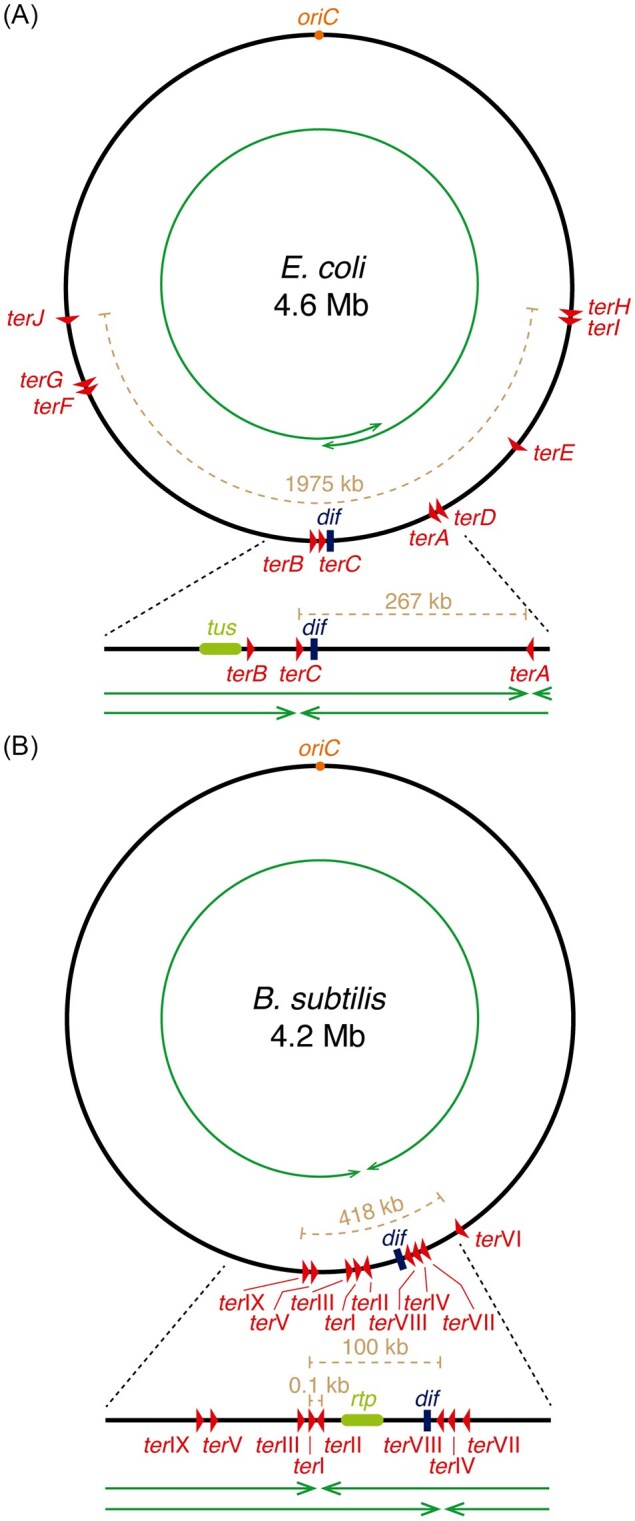
Schematic representation of replication termination in *E. coli* (A) and *B. subtilis* (B). (A, B) The black circle represents the chromosome, and the green arrowed circles indicate the direction of replication. Arrowheads representing *ter* sites show the orientation in which they block advancing replication forks (RFs). Distances between *ter* sites are indicated (dashed brown lines). If RFs progress at different speeds, solid green arrows depict the different positions where RFs may converge. Diagrams are not drawn to scale. (A) A magnified view of the innermost *ter* sites, the *dif* site, and the *tus* gene in *E. coli* is shown. *terC* is oriented to block only clockwise-moving RFs, whereas *terA* blocks anticlockwise-moving RFs. (B) A magnified view of the *B. subtilis* innermost *ter* sites, the *dif* site, and the *rtp* gene is shown. *ter*I, located only ∼0.1 kb from *ter*II, is oriented to block only clockwise-moving RFs, whereas *ter*II and/or *ter*VIII block anticlockwise-moving RFs.

In both bacteria, there are genomic terminator (*ter*) sites organized into two opposing groups: one set blocks the clockwise RF, whereas the other blocks the anticlockwise RF. Binding of the Tus protein in *E. coli*, or the RTP in *B. subtilis*, to these asymmetric *ter* sequences arrests DNA replication progression in a polar manner (Kralicek et al. 1997, Griffiths and Wake 2000, Wilce et al. 2001). Interestingly, this RF-trap mechanism is present in only a relatively small group of bacteria, and the Tus/*ter* and RTP/*ter* systems show no meaningful sequence or structural similarity, suggesting that these systems arose by convergent evolution (reviewed in Neylon et al. 2005, Goodall et al. 2023). In *E. coli*, the terminus of chromosome replication lies within a broad region flanked by the *terA* and *terC* sites, whereas in *B. subtilis* it lies within a narrow segment between the *ter*I and *ter*II or *ter*VIII sites (Fig. 3) (reviewed in Yoshikawa and Wake 1993, Rudolph et al. 2019). Notably, although Tus and RTP confine completion of DNA replication to the terminus region, they do not appear to directly participate in the termination reaction itself, as mutants lacking RTP, Tus, or *ter* sites show little phenotype and complete replication normally (Duggin et al. 2008).

Both bacteria contain a specific recombination site, *dif*, located within the terminus region close to the *terC* and *ter*VIII sites, respectively (Fig. 3). Inactivation of *dif* (deletion-induced filamentation) leads to a filamentation phenotype due to the accumulation of chromosome dimers (Kuempel et al. 1991). A site-specific recombinase (termed XerCD in *E. coli* and CodV-Rip in *B. subtilis*) is encoded in nearly all eubacterial genomes, and acts at *dif* to resolve chromosome dimers before segregation (Grainge et al. 2011).

Both model bacteria use distinct sets of nucleoid-associated proteins to organize the *ter* macrodomain. The structure of bacterial chromosomes is beyond the scope of this review, and readers are referred to recent reviews (Dame et al. 2020, Lioy et al. 2021).

### Replication termination in *E. coli*

Bioinformatic analyses suggest that binding of the Tus protein may occur at a cluster of 10 widely distributed *ter* sites (23 bp long), covering ∼43% of the chromosome (Fig. 3A). However, not all of these *ter* sites block replisomes (Duggin and Bell 2009). As the replisomes move in opposite directions on the circular genome, they ultimately converge in a head-to-head orientation at the innermost, main *terA* and *terC* sites. The *tus* gene is located near the *terC* site (Fig. 3A) (Goodall et al. 2023).

Tus binding to the asymmetric *terB*/*terC* and *terA* sites (Fig. 3A) contributes to the arrest of DnaB and Pol III, provided that the converging replisomes slow RF progression because of the accumulation of (+) supercoils between them (Elshenawy et al. 2015). The clockwise-moving RF is arrested when it encounters the first *ter* site in the non-permissive face, *terC* (Hill and Marians 1990, Neylon et al. 2005, Duggin et al. 2008, Duggin and Bell 2009). The *terC* site lies ∼180° away from *oriC*, whereas the anticlockwise-moving RF must travel an additional ∼267 kb to encounter the first non-permissive face of the Tus-*terA* complex (Duggin and Bell 2009). The near-synchronous arrival of both replisomes is crucial: if the clockwise replisome is delayed, termination may occur at *terA*, the first non-permissive site encountered by the anticlockwise RF; conversely, if the anticlockwise replisome is delayed, the clockwise replisome may bypass the non-permissive *terC* face and undergo RF arrest at the second non-permissive site, *terB* (Duggin et al. 2008, Duggin and Bell 2009).

The mechanism by which Tus-*ter* complexes block replisomes approaching from one direction but not the other has been debated for several decades, and three non-mutually exclusive mechanisms have been proposed: (i) interaction of the replicative helicase DnaB with the non-permissive face of the Tus-*ter* complex; (ii) intrinsic differences in Tus interactions at the two ends of *ter sites* allow facile dissociation of Tus by DnaB approaching from the permissive face, but not from the non-permissive face (dynamic clamp model); and (iii) helicase unwinding of the *ter* site by DnaB allows swiveling of a nucleotide (C_6_) to interact with the cytosine-binding pocket of Tus, creating a tight binding interface in which the trapped helicase prevents reannealing of the *ter* site (mouse-trap model) (Elshenawy et al. 2015, Pandey et al. 2015). Most studies suggest that the DnaB-Tus-*ter* interaction model is not the dominant mechanism underlying polar Tus-*ter* arrest (reviewed in Berghuis et al. 2018).

During exponential growth, RF fusion events occur close to the numerical midpoint of the chromosome, i.e. near the *terC* and *dif* sites, and at the same position in the presence or absence of Tus (Wendel et al. 2014, Dimude et al. 2016, Goodall et al. 2021). The RF-trap system likely prevents RFs from migrating out of the termination region, thereby controlling DNA synthesis within the RF-trap area and avoiding over-replication (Wendel et al. 2014, Dimude et al. 2015). Over-replication of the *ter* region has been observed in mutants lacking the RecG helicase, or certain exonucleases (Exo I, Exo VII) (Rudolph et al. 2013, Wendel et al. 2018, Midgley-Smith et al. 2019). Cells lacking Tus or both RecG and RnhA exhibit elevated levels of R-loops that appear to be globally distributed and drive widespread pathological over-replication across the chromosome, ultimately leading to cell lethality (Goodall et al. 2026). A Δ*tus* mutation, as well as specific *polA* mutant backgrounds result in over-replication, suggesting that Tus may facilitate Pol I take over from Pol III to fill gaps and prevent inappropriate Pol III activity (Markovitz 2005, Goodall et al. 2026). If polar arrest mechanisms fail, DnaB from one replichore may displace the leading-strand DNAP of the opposing RF and continue unwinding, albeit at low frequency. This process could generate a 3’ flap structure which, if unresolved, may lead to double-strand breaks. Double-strand break repair is beyond the scope of this review, and readers are referred to recent reviews (Ayora et al. 2011, Michel et al. 2018).

Several studies indicate a division of labor between the two type II topoisomerases: Topo II (GyrAB) removes (+) supercoils, whereas Topo IV (ParEC) relieves pre-catenanes behind the RF (Zechiedrich and Cozzarelli 1995, Hiasa and Marians 1996, Ullsperger and Cozzarelli 1996, Cebrian et al. 2015) In addition to its role in decatenation, Topo IV also contributes to chromosome organization (Zawadzki et al. 2015). Absence of Topo I (TopA) leads to extreme growth defects and accumulation of suppressor mutations, which include compensatory changes in Topo II or mutations that increase expression of *topB* (encoding Topo III, see Table 1) (Broccoli et al. 2000, Stockum et al. 2012). The role of Topo I in genome stability has been linked to the disruption of R-loops (Sutormin et al. 2022); however, R-loop accumulation does not appear to be the primary cause of the severe growth defect of Δ*topA* cells, because overexpression of *rnhA* or *recG*, which degrades and removes R-loops, respectively, does not suppress lethality (Stockum et al. 2012). Recently, it was shown that in Δ*topA* Δ*topB* mutants, Tus-dependent over-replication at the *ter* region occurs (Brochu et al. 2023). Conversely, Topo IV is required for replisomes convergence and completion of chromosome replication. Inactivation of Topo IV manifests as an inability to replicate the terminus region (Mokhtari et al. 2025), and a Topo IV cleavage hotspot has been identified near the *dif* site (Sutormin et al. 2022).

Both Topo IV (Ullsperger and Cozzarelli 1996) and, to a lesser extent, Topo III (Nurse et al. 2003, Lee et al. 2019) unlink precatenanes and catenanes to complete chromosome separation. Newly replicated chromosomes frequently require the specialized site-specific recombinase XerCD, which binds the 28-bp *dif* site to resolve chromosomal dimers, with the septal protein FtsK coordinating this dimer resolution reaction (Duggin et al. 2008, Grainge 2013, Castillo et al. 2017). It is noteworthy that if replication termination at the earlier-arriving RF is followed by replisome disassembly and replication restart before the delayed RF reaches the first *ter* site in non-permissive orientation, the innermost region -including *dif*- would be replicated twice. Whether XerCD-mediated recombination at *dif* can eliminate such “extra” DNA to preserve *terA*-*terC* integrity remains uncertain.

### Replication termination in *B. subtilis*

There are nine different *ter* sites distributed over ∼10% of the chromosome. These *ter* sites are 32-bp sequences comprising two imperfect inverted repeats, termed the A and B sites (Lewis et al. 1990). The *rtp* gene and the *dif* site map between the *ter*I/*ter*II and *ter*VIII sites (Fig. 3B) (reviewed in Yoshikawa and Wake 1993, and references therein). Notably, in *B. subtilis* strains cured of the SPβ prophage, the *ter*IX site is absent, and the *dif* locus is positioned much closer to 180° from *oriC* (Duggin 2006, Duggin et al. 2008). The exact location at which replication termination occurs is not well defined, and, as in *E. coli*, not all *ter* sites appear to block replication *in vivo* (Lewis et al. 1990, Griffiths and Wake 2000, Duggin et al. 2008). Even when positioned in the correct blocking orientation, *ter*II remains non-functional for RF arrest *in vivo* when situated at an ectopic chromosomal location (Franks and Wake 1996). Using inversion mutants that relocated the entire ∼100-kb *ter*I-*dif*-*ter*VIII region relative to *oriC* showed that, in the presence of RTP, DNA replication terminated within this region independently of its chromosomal position (Franks and Wake 1996, Kono et al. 2014). Deletion of RTP results in increased formation of chromosomal dimers in some genetic backgrounds, and the two replisomes converge at the exact position opposite *oriC* rather than at the *dif* site (Lemon et al. 2001, Kono et al. 2014).

The mechanism of polar RF arrest is not well understood (Duggin 2006, Duggin et al. 2008). Two dimeric RTPs (an unrelated functional analogue of *E. coli* Tus) bind to one *ter* site, with higher affinity for the B sub-site (Wilce et al. 2001, Vivian et al. 2007). Polar arrest of the advancing RF occurs when the B site is proximal to the approaching RF (Smith and Wake 1992). Peptides fused to the C-terminus of RTP reduce the efficiency of RF arrest *in vivo* without altering DNA binding *in vitro*, supporting a RF-trap mechanism through protein-protein interactions (Duggin 2006). Another study proposed that asymmetrical features of the structure of RTP-C110S in complex with the B site of the *terI* terminator underlie the mechanism of polar RF arrest at the complete terminator site (Vivian et al. 2007).

*Bacillus subtilis* possesses two type I [TopA (Topo I) and TopB (Topo III)] and two type II [GyrAB (Topo II), and ParEC (Topo IV)] topoisomerases, but their specific contributions to replication termination remain poorly defined. The essentiality of TopA enzyme, which removes (−) supercoils, can be suppressed by overexpression of ParEC, but not by overexpression of TopB (Reuss et al. 2019). Indeed, both type I DNA topoisomerases are dispensable in ParEC-overexpressing cells. The topological stress generated by converging RFs cannot be processed by the TopB enzyme. In fact, *in vitro*, the *Bacillus* TopBβ enzyme partially removes (−) supercoils but lacks decatenase activity (Li et al. 2006).

Finally, newly replicated genomes require the specialized site-specific recombinase RipX-CodV (*a.k.a*. XerD-XerC) acting at *dif* to resolve dimeric chromosomes, in concert with the SftA or SpoIIIE translocases (Sciochetti et al. 2001, Kaimer et al. 2011). This recombination step, together with ParEC-mediated decatenation, is essential for completion of chromosome segregation.

Inactivation of RTP increases the formation of chromosomal dimers in cells lacking RipX (Lemon et al. 2001). Genetic analyses revealed that a Δ*rtp* Δ*ripX* double mutant strain exhibits a >200-fold increase in anucleate cell accumulation, a phenotype that is markedly alleviated by *recA* inactivation. In the Δ*rtp* Δ*ripX* Δ*recA* triple mutant, only a >10-fold increase in anucleate cells is observed, suggesting that the defect primarily arises from homologous recombination (Lemon et al. 2001, Sanchez et al. 2007, Goodall et al. 2023). We propose that the absence of RTP leads to over-replication and an increased frequency of crossover events, generating chromosomal dimers that cannot be resolved in the absence of RipX. Mutants lacking the RecG helicase severely over-replicate the *ter* region and display similar levels of anucleate cells as Δ*ripX* cells (Sanchez et al. 2007, S.A. personal communication). Furthermore, the RipX subunit of the site-specific recombinase is required for unloading translocating SMC complexes upon their arrival at the *ter* region (Karaboja et al. 2021), although whether this process is related to the completion of DNA replication remains unclear.

### Replication control

The correct coordination of DNA replication with cellular growth is crucial in both model bacteria. Distinct mechanisms ensure tight control of the different steps of DNA replication. Pre-initiation regulation and timely genome partitioning during cell division are beyond the scope of this review; readers are referred to recent reviews (Jameson and Wilkinson 2017, Katayama et al. 2017, Reyes-Lamothe and Sherratt 2019, Gogou et al. 2021, Grimwade and Leonard 2021, Cameron and Margolin 2024).

First, regulation of replication initiation is fundamental to cell growth and cell cycle control: over-initiation leads to genome instability, whereas under-initiation results in fewer incidents of RF stress. To commit to replication initiation and ensure that genome duplication occurs only once per cell cycle, both model bacteria use different sets of regulators. In *E. coli*, the identified regulators include proteins (DiaA, Hda, Dam methylase, SeqA, IHF, and Fis) and several chromosomal loci located outside *oriC* (*datA*, and the DnaA-reactivating sequences *DARS1* and *DARS2*) that regulate the availability of DnaA·ATP and control the switch between the active DnaA·ATP and the inactive ADP-bound state (Kaguni 2006, Katayama et al. 2017, Dewachter et al. 2018). *datA*, in concert with IHF, promotes hydrolysis of DnaA·ATP to DnaA·ADP (Kasho et al. 2017), and *DARS1* and *DARS2* convert DnaA·ADP to the apo-form, which rapidly binds ATP, present at high intracellular concentrations, thereby generating DnaA·ATP (Fujimitsu et al. 2009). DiaA interacts with and promotes DnaA·ATP assembly at DnaA boxes to ensure timely initiation, while competing with DnaB for interaction with DnaA and thereby indirectly exerting a negative effect on DnaB loading (Keyamura et al. 2009). SeqA and Hda negatively control replication to prevent over-initiation through distinct mechanisms (Boye et al. 1996). Before initiation, the 5'-GATC-3' Dam sites on both strands within *oriC* are fully methylated by Dam methylase, and SeqA binds poorly to fully methylated sites (Slater et al. 1995). After initiation, DnaA·ADP dissociates from *oriC*, allowing SeqA to bind hemi-methylated Dam sites. In this context, SeqA prevents DnaA·ATP from binding to low affinity DnaA-boxes while permitting binding to higher-affinity DnaA recognition sites on hemi-methylated *oriC* (Nievera et al. 2006). Once the hemi-methylated nascent strands of *oriC* become fully methylated by Dam methylase, SeqA-mediated sequestration ends, rendering *oriC* available for the next replication cycle (Slater et al. 1995).

Hda·ADP, upon interaction with DNA-bound DnaN/β sliding-clamp, interacts with DnaA·ATP and stimulates ATP hydrolysis to generate inactive DnaA·ADP in proportion to chromosome number, thereby coordinating the transition from initiation to elongation of DNA replication (Katayama et al. 1998, Su’etsugu et al. 2008, Knoppel et al. 2023). This is consistent with the over-initiation phenotype and growth defects observed in Δ*hda* cells (Knoppel et al. 2023).

A strain lacking *DARS1, DARS2, datA*, and *hda*, displays near wt behavior in minimal medium (non-overlapping replication cycles), whereas under rapid growth (overlapping replication cycles), these cells exhibit initiation instability at an almost constant DnaA concentration during cell elongation (Boesen et al. 2024).

In *B. subtilis*, six DnaA-box clusters are found outside *oriC*, and deletion of these clusters (Δ6 strain) results in early initiation, suggesting that replication initiation is sensitive to the intracellular level of free DnaA (Okumura et al. 2012). However, over-initiation of replication was not observed when *B. subtilis* DnaA was ∼10-fold overproduced in otherwise wt cells, in contrast to the effect observed in *E. coli* (Moriya et al. 1990). Remarkably, *B. subtilis* lacks homologues of DiaA, Hda, Dam, SeqA, and IHF, and the *datA, DARS1*, and *DARS2* loci are absent. Whether *B. subtilis* possesses regulators that balance DnaA·ADP/DnaA·ATP interconversion remains unknown. Instead, DnaA activity is regulated through direct interactions with several proteins that affect DnaA cooperative binding and oligomerization at *oriC*, including YabA, Soj (*a.k.a*. ParA), DnaD, CcrZ, and SirA (Hayashi et al. 2005, Wagner et al. 2009, Bonilla and Grossman 2012, Scholefield et al. 2012, Scholefield and Murray 2013, Gallay et al. 2021). YabA, Soj, and DnaD interact with and inhibit DnaA oligomerization *in vitro* (Scholefield et al. 2012, Scholefield and Murray 2013). YabA, which interacts with both DnaA and DnaN, associates with the replisome and stimulates dissociation of DnaA from *oriC*, an effect regulated by DnaN (Hayashi et al. 2005, Noirot-Gros et al. 2006, Goranov et al. 2009, Merrikh and Grossman 2011). Fluorescence microscopy has shown that DnaA oligomerization at *oriC* occurs multiple times during the cell cycle, a process counteracted by YabA and Soj to prevent over-initiation (Schenk et al. 2017). Soj (ParA), together with Spo0J (ParB), belongs to a conserved protein family required for efficient plasmid and chromosome partitioning in many bacterial species.

DnaD inhibits cooperative binding of DnaA·ATP to DNA *in vitro* without affecting nucleotide exchange, suggesting that DnaD may act as a negative regulator of DnaA·ATP (Bonilla and Grossman 2012). However, DnaD is also required for both replication initiation and replication restart. The negative regulatory activity of DnaD may be modulated by other pre-primosomal proteins or by DnaD abundance. During sporulation, GFP-SirA accumulates at the replisome, and SirA may prevent DnaA·ATP accumulation at *oriC via* direct protein-protein interaction (Wagner et al. 2009, Jameson et al. 2014, Matthews and Simmons 2019). Furthermore, SirA blocks DnaD interaction with DnaA·ATP thereby restricting helicase recruitment to *oriC* during sporulation to further inhibit initiation events (Winterhalter et al. 2023). The CcrZ kinase, which interacts with DnaA and DnaB, acts as a positive regulator of replication initiation (Wozniak et al. 2022). Inactivation of *ccrZ* causes under-initiation, whereas *ccrZ* overexpression or *yabA* deletion leads to over-initiation (Goranov et al. 2009, Merrikh and Grossman 2011, Gallay et al. 2021, Reed et al. 2025). CcrZ also interacts with FtsZ, thereby coupling replication with cell division (Gallay et al. 2021).

Second, in both bacteria, DnaA also functions as a transcription factor, establishing a highly conserved homeostatic feedback loop that controls expression of the *dnaA*-*dnaN* operon (Menikpurage et al. 2021). In *E. coli*, DnaA·ATP bound to specific 6-mer boxes fully represses transcription of the *dnaA*-*dnaN* operon from upstream promoters, whereas binding of DnaA·ATP to its cognate 9-mer DnaA-box triggers replication initiation (Atlung et al. 1985, Speck et al. 1999). The nucleotide-bound state of DnaA may regulate its transcriptional activity, *e.g*. of the *nrdAB* genes, which encode a nucleotide reductase required for deoxyribonucleotide biosynthesis. High levels of DnaA·ATP repress *nrdAB* transcription, whereas DnaA·ADP or low levels of DnaA·ATP bound to high-affinity DnaA boxes can activate *nrdAB* transcription without altering the timing of *nrdAB* expression (Menikpurage et al. 2021). Indirectly, the DnaN/β sliding-clamp, by stimulating ATP hydrolysis by DnaA, may modulate DnaA-mediated gene regulation (Katayama et al. 1998). A comprehensive analysis of the impact of DnaA’s transcriptional activity on replication initiation in *E. coli* remains to be performed.

In *B. subtilis*, DnaA binds not only to cognate DnaA boxes in the promoter region of *dnaA* -repressing the *dnaA*-*dnaN* operon-, but also binds to other regions (Ogura et al. 2001, Ishikawa et al. 2007). Upon inhibition of DNA replication, expression of 71 genes that regulate lifestyle transitions was altered independently of RecA, and their promoters contained at least two potential DnaA-binding sites (Goranov et al. 2005).

*In vivo* ChiP-seq analysis confirmed stable binding of DnaA to at least eight regions (Ishikawa et al. 2007), whereas *in vitro* whole-genome binding assays with purified DnaA revealed binding to 269 regions and showed that the nucleoid-associated protein Rok influences DnaA binding *in vivo* (Smith and Grossman 2015). In these experiments, overall binding patterns for DnaA·ADP were similar to those for DnaA·ATP, although most DnaA cognate sites displayed slightly higher affinity for DnaA·ATP, and a few regions (*e.g*. the *sda* promoter) showed strong preference for DnaA·ATP (Smith and Grossman 2015). Controlled expression of the *dnaA*-*dnaN* operon is also mediated by the antisense RNA *arrA*. Transcription of *arrA* is repressed by DnaA, and loss of *arrA* transcription increases cellular levels of DnaA and DnaN (Sedivy et al. 2025).

Third, both model bacteria, which initiate replication at a fixed cell mass, can adjust their growth rates efficiently in response to an increase in cell mass by employing similar strategies that coordinate cell size and the cell cycle (Donachie 1968, Sauls et al. 2019). In nutrient-rich media (e.g. LB), new rounds of DNA replication begin before previous rounds are completed, resulting in a doubling time shorter than the time required to replicate the chromosome and in a variable number of initiation events per cell cycle (Yoshikawa et al. 1964). Under nutrient-limited conditions, both bacteria initiate replication once per cell cycle, completing replication within the same generation in which it was initiated (Cooper and Helmstetter 1968).

Both *E. coli* and *B. subtilis* have evolved remarkably divergent strategies to regulate replication onset in response to metabolic state. Certain nutrient limitations, such as amino acid starvation, trigger the stringent stress response, increasing levels of guanosine tetra- and pentaphosphate second messengers [collectively known as (p)ppGpp]. In *E. coli*, elevated (p)ppGpp, which directly interacts with RNAP, arrests replication (reviewed in Gourse et al. 2018, Hallgren and Jonas 2023). The stringent response reduces DnaA protein levels, either by decreasing *dnaA* transcription through changes in DNA superhelicity at *oriC*, or by degrading inactive DnaA·ADP in a process activated by polyphosphate (Kraemer et al. 2019, Gross and Konieczny 2020). Notably, (p)ppGpp fails to arrest replication initiation following expression of a hyperactive DnaA mutant that mimics DnaA·ATP (Kraemer et al. 2019). Furthermore, the replication elongation rate is modestly affected by (p)ppGpp independently of transcription, possibly through inhibition of the *E. coli* primase, which is inhibited by ppGpp *in vitro* (Denapoli et al. 2013, Maciag-Dorszynska et al. 2013). In contrast, replication initiation in *B. subtilis* is insensitive to (p)ppGpp accumulation. In this bacterium, (p)ppGpp inhibits enzymes required for GTP biosynthesis and does not bind RNAP (Gourse et al. 2018, Bange and Bedrunka 2020). (p)ppGpp directly interacts with the nucleotide-binding site of DnaG and inhibits its activity in a dose dependent manner (Wang et al. 2007, Rymer et al. 2012, Giramma et al. 2021), inducing a reversible RF arrest that is independent of the SOS response (Gándara and Alonso 2015). Inhibition of DnaG, together with significantly reduced GTP levels, likely contributes to halting replication elongation and preserving genome integrity during nutrient limitation (Wang et al. 2007, Denapoli et al. 2013).

Central metabolism differentially regulates replication in the two bacteria (reviewed in Soultanas and Janniere 2023, and references therein). In *E. coli*, two metabolites (acetyl-CoA and acetyl-phosphate) facilitate DnaA acetylation, which prevents the formation of the active DnaA·ATP-*oriC* nucleoprotein complex, whereas CobB, acting as a deacetylase, facilitates replication initiation (Zhang et al. 2016). In response to environmental stress, polyphosphate interacts with the CobB deacetylase and inhibits its activity, thereby precluding the initiation of DNA replication (Boguszewska et al. 2026). In contrast, in *B. subtilis*, replication initiation seems not to be under the control of acetylation whereas several catabolic enzymes directly interact with the primosomal proteins DnaG, DnaC, and DnaE, thereby slowing replication elongation in response to environmental changes (Horemans et al. 2022, Soultanas and Janniere 2023). Divergent evolutionary trajectories likely underlie the substantial differences in how these two bacteria adjust their replication rates in response to nutrient limitation.

### Replication re-initiation

Replisome progression is challenged at different frequencies in both model bacteria by encounters with barriers, such as damaged DNA templates, or by RTCs, which lead to varying degrees of replication stress and responses. In the wt background, replisome pausing at highly transcribed *rrn* loci, with subsequent disassembly, is observed in *B. subtilis*, whereas the sites of slow velocity oscillations observed in *E. coli* are not coincident with *rrn* loci (Mangiameli et al. 2017b, Huang et al. 2023). In contrast, an artificial reduction in the number of *rrn* loci significantly increased RTCs, causing a greatest growth defect, primarily due to high mortality rates, in *E. coli* than in *B. subtilis* cells (Fleurier et al. 2022, Fan et al. 2023). These results suggest that both bacteria exhibit different responses to RTCs and subsequently different mechanisms of replication restart. In *E. coli*, the physical coupling between the leading ribosome and the elongating RNAP core enzyme helps maintain productive transcription-translation coupling, prevents RNAP backtracking, and reduces the likelihood of R-loop formation (Nudler 2012). Conversely, in *B. subtilis*, transcription and translation are uncoupled, allowing ribosome-free RNAP to backtrack and nascent RNAs are more prone to forming hairpins and R-loops (Johnson et al. 2020).

Replication re-initiation may involve re-priming events mediated by pre-primosomal proteins, which are not part of the elongating replisome (reviewed in Marians 2000, Windgassen et al. 2018, Carrasco et al. 2024, and references therein). Despite their fundamental role in replication re-initiation at sites other than *oriC*, pre-primosomal proteins are poorly conserved between the two bacteria, with the exception of PriA (Blaine et al. 2023, Torres et al. 2025). Furthermore, pre-primosomal proteins are partially dispensable in *E. coli* but essential in *B. subtilis* (reviewed in Bonde et al. 2024, Carrasco et al. 2024).

Finally, in *E. coli*, the selection of pre-primosomal proteins utilized during replication restart depends on the nature of the accumulated DNA structure. For simplicity, three main types of replication restart substrates will be considered: i) a stalled fork structure resembling a leading-strand template lesion, with a gap on the leading strand and a fully synthesized lagging strand, termed a 5'-fork-like DNA structure; ii) a stalled fork structure mimicking a lagging-strand template lesion, with a gap on the lagging strand and fully synthesized leading strand, termed a 3'-fork-like DNA structure; and iii) an abandoned replication fork, mimicking a RF disassembled at a roadblock, with fully synthesized leading and lagging strands, termed a replicated fork structure or 3'-5'-fork (Michel and Sandler 2017). In contrast, in *B. subtilis*, the PriA-DnaD-DnaB pre-primosomal complex is utilized for replication restart independently of the nature of the accumulated DNA structure.

### Replication restart in *E. coli*

When a replisome encounters spontaneous (e.g. an oxidized nucleobase) or exogenous (bulky or non-bulky lesion) threats on the lagging-strand template, the damage can be efficiently bypassed provided the helicase does not disassemble (Heller and Marians 2006b). In this scenario, DnaB, which is highly stable, remains bound to the lagging-strand template, facilitating reloading of DnaG, and repriming. The stalled lagging-strand replicase is released and recycles to the next primer synthesized at the lagging strand. Alternatively, the slow-moving DnaB interacts with and recruits a new cytosolic Pol III core enzyme to re-establish the full replisome following reloading of the DnaN/β sliding-clamp. Both mechanisms leave a lesion-containing gap behind (Pagès and Fuchs 2003, McInerney and O’Donnell 2004, Yeeles and Marians 2011).

When the Pol III core enzyme encounters a lesion in the leading-strand template, such as a single cyclobutane pyrimidine dimer, it may transiently stall, but remain transiently associated. DnaB continues unwinding, and the leading-strand DNAP recycles to a new primer synthesized on the leading strand by the DnaG primase (lesion skipping mechanism) (Yeeles and Marians 2011, Yeeles and Marians 2013). Alternatively, the replisome disassembles, and reloading of a DnaBC complex must occur in a sequence-independent manner outside *oriC*. This process requires pre-primosomal proteins different from those involved in loading the two DnaBC complexes at *oriC via* DnaA·ATP (reviewed in Michel and Sandler 2017, Windgassen et al. 2018, and references therein). With the exception of PriA, the pre-primosomal proteins identified as participants in this process (PriB, PriC, DnaT, and Rep) are not widely conserved outside the γ-Proteobacteria Class (Sandler 2000). Replication restart can proceed through multiple pathways, with PriA, PriB, PriC, DnaT, and Rep proteins contributing to multiple pathways that recognize abandoned and stalled RFs (Sandler 2000).

Different combinations of mutants and the identification of suppressor mutations have helped test the “multiple replication restart pathways model” (Sandler 2000). Genetic analyses revealed that *in vivo*, the absence of Rep or PriC results in synthetic lethality in a Δ*priA* or Δ*priB* background (reviewed in Michel and Sandler 2017). The growth defect of Δ*priA* is suppressed by mutations in the *dnaC* gene, such as *dnaC*810. DnaC810 loads the DnaB replicative helicase onto SSB-ssDNA complexes in the absence of PriA (Xu and Marians 2000). The gain-of-function mutant *dnaC*809 *dnaC*820 suppresses the effect of the absence of PriA, PriB, and weakly of *priB pri*C mutant cells (Sandler et al. 1999, Sandler 2000). Additionally, the *dnaC*824 mutation efficiently suppresses the phenotype of *priB rep* and *priB priC* strains but only weakly suppresses the requirement for PriA (Sandler et al. 1999, Boonsombat et al. 2006). *In vitro* systems using purified proteins and specific DNA substrates that mimic different types of stalled/abandoned RFs were subsequently used to test these pathways (Heller and Marians 2005a,2005b, Heller and Marians 2006a, Yeeles and Marians 2011, Yeeles and Marians 2013).

Live-cell studies revealed that DnaB is highly stable at RFs, and PriA forms spontaneous foci that co-localize with replisome markers in ∼7% of wt unperturbed cells (Beattie et al. 2017, Soubry et al. 2019). Under environmental stress, however, >70% of cells exhibit PriA foci that colocalize with replisome markers (Soubry et al. 2019), suggesting that PriA is the primary contributor to DnaB reloading at the abandoned RFs after UV-induced DNA damage. PriA may prevent DNAP progression until DnaB is assembled (Yuan and McHenry 2009). Is PriA the first responder to replication stress or genetic redundancy exists with certain structure-specific preferences? *In vitro* reconstitution assays using purified proteins and substrates that mimic different types of stalled fork structures (3'-forks, 5'-forks, and replicated fork structures) have been employed to address this question.

In the first pathway, PriA acts as the central protein together with PriB and DnaT to promote reassembly of the DnaBC complex (at 3'-forks or abandoned RFs), followed by release of the accessory pre-primosomal proteins (Manhart and McHenry 2013, Manhart and McHenry 2015). PriA binding preferences among different stalled fork structures remain controversial. Some reports indicate preferential binding to 3’-forks, whereas others show ∼two-fold higher affinity for 5'-fork DNA compared to 3'-fork DNA (Nurse et al. 1999, Tanaka et al. 2007, Wang et al. 2020). These discrepancies can be attributed to the differences in the length of the ssDNA region used in the different studies. PriA is an SF2 3'→5' DNA helicase that unwinds the nascent lagging strand at abandoned RFs to provide a site for DnaB loading (Lee and Marians 1987, Jones and Nakai 2001, Lopper et al. 2007). PriA alone cannot unwind 3'-fork DNA; it requires SSB and PriB that stimulate its helicase activity (Cadman and McGlynn 2004, Cadman et al. 2005). SSB, which interacts with pre-primosomal proteins such as PriA, PriB, and PriC, may bind the ssDNA region at stalled/abandoned RFs (Bonde et al. 2024). PriA interaction with SSB stabilizes the SSB_35_ binding mode over the SSB_65_ mode, leading to the formation of a PriA-SSB-DNA ternary complex in which SSB is excluded from a region of ssDNA that becomes available for PriB and DnaT binding (Bhattacharyya et al. 2014, Manhart and McHenry 2015).

Biochemical and structural studies using model RFs have been crucial for defining pre-primosomal protein binding sites and the conformational changes occurring during assembly. PriB binds tightly to the PriA-DNA complex (Lopper et al. 2007, Duckworth et al. 2023). PriB additionally interacts with DnaT, recruiting it to the PriA-PriB-DNA nucleoprotein complex. Interaction between PriB and DnaT promotes pre-primosome release and subsequent loading of the DnaBC complex (Lopper et al. 2007, Manhart and McHenry 2013, Manhart and McHenry 2015). This PriA-PriB-DnaT mechanism is likely the predominant pathway for replication restart at 3'-fork and abandoned RF DNA substrates and has been reconstituted *in vitro* (reviewed in Windgassen et al. 2018).

In the second pathway, PriC efficiently recognizes 5'-fork-like intermediates (Heller and Marians 2005a) and interacts with and displaces SSB from the template leading-strand gap (Wessel et al. 2013). At abandoned RFs, PriC is sufficient to load the DnaBC complex (Heller and Marians 2005a). At 5'-forks, PriC may load the Rep helicase onto the lagging-strand template, facilitating Rep-mediated displacement of the nascent lagging strand (Heller and Marians 2006a, 2007, Yeeles and Marians 2011, Yeeles and Marians 2013). Despite extensive study, the *in vivo* determinants governing selection of the PriC-Rep pathway remain largely unresolved. A third pathway, involving PriA-PriC-DnaT, remains less well understood, as it has not been reconstituted *in vitro*. Notably, the 3'→ 5' DNA helicase activity of PriA is required for this pathway (Michel and Sandler 2017, Windgassen et al. 2018).

Dysregulation of replication restart leads to genomic instability (Rudolph et al. 2013). How is replication restart regulated? In the absence of an active DnaB helicase assembled on the lagging strand, PriA acts as a checkpoint by blocking the intrinsic strand-displacement activity of the Pol III HE on the complementary strand (Xu and Marians 2003, Yuan and McHenry 2009, Manhart and McHenry 2013). However, little is known about whether PriA-dependent loading of the replicative helicase is regulated.

### Replication restart in *B. subtilis*

Live-cell studies revealed that the replisome undergoes spontaneous disassembly ∼5 times per cell cycle, followed by replisome reassembly once replication stress is resolved (Merrikh et al. 2012, Mangiameli et al. 2017b, Huang et al. 2023). These observations suggest that DnaC association with ssDNA frequently encounters obstacles, as ∼40% of unperturbed cells contain only a single replisome. Indeed, transcription inhibition increases the lifetime of DnaC-DNA complexes (Mangiameli et al. 2017b). Sequence-independent initiation is therefore essential to restart replication at sites where it has failed.

In this bacterium, PriB, PriC, DnaT, and Rep restart proteins are absent, and PriA is essential. Except for PriA, reloading of a single DnaC-DnaI complex at leading- or lagging-strand stalled forks utilizes a set of essential pre-primosomal proteins similar to that involved in *oriC*-dependent initiation (see above). PriA, which binds branched intermediates, interacts with DnaD and DnaB promoting their loading to form a pre-primosome complex. Indeed, depletion of PriA for roughly one doubling time prevents replication restart after spontaneous replisome disassembly in ∼90% of unperturbed cells (Mangiameli et al. 2017b). PriA may travel with the replisome in unperturbed cells through its interaction with SsbA, as PriA foci formation is significantly reduced in *ssbA*ΔC35 cells (Costes et al. 2010). Alternatively, SsbA bound to ssDNA at stalled forks interacts with and recruits PriA in response to replication stress (Lecointe et al. 2007).

PriA facilitates replication restart by recognizing and binding branched intermediates that arise when RFs stall. *In vitro*, PriA binds abandoned RF, 5'-fork-like, and 3'-fork-like structures with similar affinity (Marsin et al. 2001). PriA exhibits ssDNA dependent ATPase activity that is inhibited by SsbA (Polard et al. 2002). DnaD physically interacts with PriA and with DnaB and stimulates DnaB binding to unreplicated forks and 3’-fork structures (Marsin et al. 2001, Bruand et al. 2005).

The pre-primosome complex, in concert with DnaI, loads the DnaI-DnaC complex or assembles a DnaC hexamer on the lagging strand (Masai et al. 1999, Marsin et al. 2001, Polard et al. 2002, Bruand et al. 2005). A fully reconstituted unidirectional *in vitro* replication system using a 3'-fork DNA substrate demonstrated that PriA, DnaD, and DnaB, together with DnaI, are necessary and sufficient for loading DnaC onto the lagging-strand template (Sanders et al. 2010, Seco et al. 2013).

RecA, beyond its canonical role in DNA strand exchange, regulates recruitment of pre-primosomal proteins to stalled forks (Vlasic et al. 2014, Million-Weaver et al. 2015b). *In vitro*, RecA inhibits/delays PriA-dependent replication restart by binding the ssDNA region, in a process facilitated by RecO without affecting replication elongation (Vlasic et al. 2014). The physiological relevance of this inhibition remains to be determined. *In vitro*, the 5'→3' branch migration translocase RecD2 counteracts the negative effect of RecO and RecA on PriA-dependent replication restart by promoting active RecA disassembly from the template strand (Ramos et al. 2022). Through its strand-switching activity, RecD2 may help remove obstacles from template strands (Hormeño et al. 2025). Consistently, RFs collapse more frequently in Δ*recD2* cells (Walsh et al. 2014). PcrA, an essential helicase (Petit et al. 1998), is important for replication of highly transcribed regions (Merrikh et al. 2015). Notably, PcrA also facilitates RecA disassembly from the template strand *in vitro* (Carrasco et al. 2022).

Is replication restart temporally regulated? In addition to RecA, other recombination proteins may contribute to timely PriA-dependent loading of DnaC. Live-cell fluorescence microscopy studies revealed that in unperturbed growing cells, several recombination proteins may travel with RFs, including RarA, RecQ, RecS, and RecG (Lecointe et al. 2007, Costes et al. 2010), or be assembled by SsbA at stalled RFs. Notably, RecA interacts with both its positive (RarA) and negative (PcrA and RecD2) modulators (Romero et al. 2019, Ramos et al. 2022). Using a 3'-fork DNA substrate, RarA, similar to RecA, was shown to restrict PriA-dependent replication restart (Carrasco et al. 2018). This inhibition appears initiation-specific rather than a nonspecific consequence of RF binding, as replication elongation remains unaffected (Carrasco et al. 2018). Furthermore, the DisA protein, which also binds branched DNA intermediates, neither affects PriA-dependent re-initiation nor replication elongation (Raguse et al. 2017). The molecular basis of RarA contribution to replication restart remains elusive.

Can replication re-initiation proceed in the absence of PriA? A DnaB S371P mutant, which exhibits increased interaction with DnaI, is capable of loading a DnaI-DnaC complex at stalled forks in Δ*priA* mutant cells (Bruand et al. 2001, Rokop et al. 2004). The precise mechanism by which DnaB S371P, together with DnaI, promotes DnaC loading at abandoned RFs remains unresolved.

## Conclusions and future perspectives

A comprehensive understanding of DNA replication in different bacterial species is required to fully grasp bacterial proliferation, with intriguing implications across multiple research fields. These include the use of bacterial species and the modification of their metabolic pathways through molecular biology techniques for the production or degradation of a huge variety of compounds, as well as for the identification of novel targets to combat antibiotic resistance.

DNA replication has been extensively studied in both *E. coli* and *B. subtilis* using diverse experimental approaches, including genetic analyses, microscopy studies, biochemical assays with different *in vitro* reconstituted replication systems and substrates, single-molecule techniques, and structural studies. While the essential processes of replication initiation, bidirectional DNA synthesis, and termination are broadly conserved, these studies have revealed that distinct molecular machines and specific mechanisms for DNA synthesis have evolved to adapt each model bacterium to its environment. The mechanisms of replication re-initiation following replication stress have also been investigated, but remain less well understood.

In this review, we have described the similarities and differences between both model bacteria at the level of the proteins involved and the mechanisms employed. Specifically, we have discussed factors affecting replication initiation, such as the presence of a bipartite *oriC* in *B. subtilis* and a single *oriC* region in *E. coli*, the distinct regulatory mechanisms governing DnaA activity, the different strategies for replicative helicase loading, and the divergent priming mechanisms for DNA synthesis, in both bacteria. At present, it remains unclear why *B. subtilis* and other Firmicutes use an RNA-DNA hybrid primer, as eukaryotic cells do, whereas *E. coli* can extend an RNA primer during chromosome replication, as bacteriophages do (O’Donnell 2006, McHenry 2011).

The distinct assembly and composition of the replisomes, including their different number of subunits, may also explain the alternative solutions adopted by each bacterium in response to replication stress. Current evidence suggests that *E. coli* preferentially skips lesions, leaving a gap behind the replisome that is repaired in a post-replication manner by error-free DDT sub-pathways, or, if these fail, by error-prone DDT sub-pathways (Cox et al. 2023). In contrast, *B. subtilis* remodels stalled forks to circumvent lesions/barriers, or rarely bypasses lesions at stalled forks through distinct error-prone DDT sub-pathways before restarting DNA replication (Carrasco et al. 2024). Replisome reloading in *B. subtilis* appears to rely exclusively on the PriA-DnaD-DnaB pre-primosome complex, whereas *E. coli* employs multiple restart pathways -some involving PriA, some not. Finally, the genomic distribution of *ter* sites, and the conflicts that cause replisome slowing, differ between both bacteria. DNA replication termination has been extensively studied in *E. coli*, but remains poorly understood in *B. subtilis*.

Despite significant progress, many questions remain unanswered and warrant further investigation. For instance, it will be of great interest to elucidate the precise molecular mechanisms by which proteins control the timing of replisome disassembly and reassembly ahead of lesions in *E. coli* or at stalled forks in *B. subtilis*. Moreover, the mechanisms underlying PriA-mediated replication restart also remain incompletely defined. Advanced fluorescence live-cell imaging, single-molecule analyses, and structural studies of the relevant protein complexes will likely provide important insights into these fundamental processes.
